# Graph-Based Feature Selection Approach for Molecular Activity Prediction

**DOI:** 10.1021/acs.jcim.1c01578

**Published:** 2022-03-22

**Authors:** Gonzalo Cerruela-García, José Manuel Cuevas-Muñoz, Nicolás García-Pedrajas

**Affiliations:** Department of Computing and Numerical Analysis, University of Córdoba, Campus de Rabanales, Albert Einstein Building, E-14071 Córdoba, Spain

## Abstract

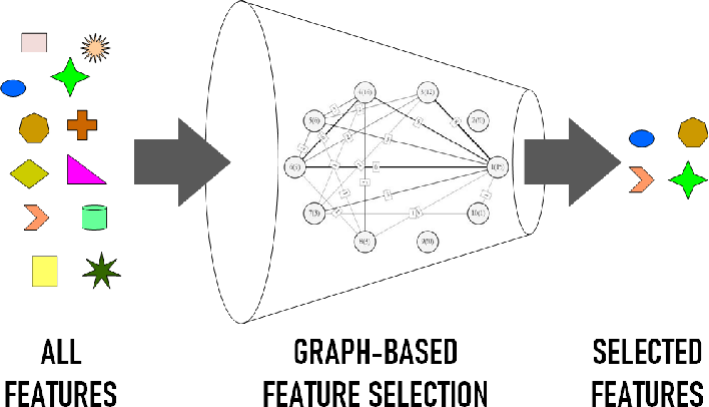

In the construction of QSAR models for the prediction of molecular activity, feature selection is a common task aimed at improving the results and understanding of the problem. The selection of features allows elimination of irrelevant and redundant features, reduces the effect of dimensionality problems, and improves the generalization and interpretability of the models. In many feature selection applications, such as those based on ensembles of feature selectors, it is necessary to combine different selection processes. In this work, we evaluate the application of a new feature selection approach to the prediction of molecular activity, based on the construction of an undirected graph to combine base feature selectors. The experimental results demonstrate the efficiency of the graph-based method in terms of the classification performance, reduction, and redundancy compared to the standard voting method. The graph-based method can be extended to different feature selection algorithms and applied to other cheminformatics problems.

## 1 Introduction

In the construction of quantitative structure–activity relationship (QSAR) models based on classification or regression techniques, the preprocessing step is a fundamental component to avoid the use of data that yield an identical effect, no effect, or even a deceptive effect.^1^ Feature selection is one of the most common tasks used in this preprocessing step. The selection of an optimal set of features from which a model can achieve maximum performance is a nondeterministic polynomial problem (NP).

The objective of feature selection is to eliminate, as much as possible, the amount of irrelevant and/or redundant features to improve the performance of the prediction algorithms, reducing the negative effects related to high dimensionality, accelerating the learning process, and improving the generalization and interpretability of models. Feature selection methods can rank individual features according to their importance (ranking methods) or evaluate complete sets of features to select an optimal subset (feature subset selection methods). This paper is only concerned with the latter.

From a taxonomic point of view, feature selection methods are traditionally divided into four categories: (i) filter methods, (ii) wrapper methods, (iii) embedded methods, and (iv) hybrid methods.^2^ Filters methods select the features regardless of the algorithm used in building the model. A large number of filter methods have been described in the literature, and among the most used are the following: information gain,^3^ gain ratio,^4^ minimum redundancy, maximum relevance,^5^ Chi-square,^4^ fast correlation-based filter,^6^ correlation-based feature selection,^4^ Fisher score,^7^ fast clustering-based feature selection (FAST),^8^ and Relief or ReliefF.^9^

Wrappers methods choose the optimal subset of features for evaluating the performance of the modeling algorithm as if it were a black box. Wrappers require a higher computational cost compared to the filter methods. Furthermore, the subsets of features are biased toward the modeling algorithm used in the evaluation. For this reason, the use of independent validation samples is necessary for the reliable estimation of the error.

Embedded methods integrate the selection of features within the modeling algorithm, either as part of the predictive/descriptive method or as an extended functionality, and thus, the selection of features is accomplished during the execution of the modeling algorithm.

Hybrid methods combine the advantages of filter and wrapper methods. Usually these methods initially apply a filter method to reduce the number of features, obtaining in many cases several possible subsets. A wrapper method is then used to obtain the best subset of features.

Previous literature on the construction of QSAR models shows that the most used feature selection methods are the following: chi-square (CS),^10,11^ gain ratio (GR),^12,13^ information gain (IG),^14,15^ unbalanced correlation score (UCS),^7^ mutual information (MI),^16,17^ standard correlation score (Fisher score, FS),^18,19^ F-score (FS) base ranking,^20−22^ Shannon entropy (SE),^23,24^ recursive feature elimination (RFE),^25−28^ and the fast clustering-based feature selection algorithm (FAST).^29^

Ensemble approaches, based on bagging and/or boosting, have been proposed for feature selection.^30^ These methods have two configurable components: the boosting scheme and the base feature selection algorithm to be used. Ensembles of feature selectors are constructed by repeatedly applying feature selection algorithms and then combining their results. Ensembles of feature selectors focused on overcoming class imbalance problems have also been proposed.^31^

In the construction of feature selection ensembles, the combination of the results of the different base selectors is crucial.^32^ The set of methods for combining feature subset selectors is usually limited to take into account the result of applying each feature selector by storing in a vector the number of times that each feature was selected; this vector is used to obtain the final selection. The most straightforward methods are the intersection or union of feature sets. However, both of them produce poor performance. The intersection often returns very small sets and thus results in poor performance. The union generally achieves better performance, but with the drawback of almost negligible reduction. A more efficient solution is to use a vote threshold to obtain sets of features based on the classification performance^30^ or subject to a data complexity measure.^33^

The main drawback of these three approaches is that they disregard relationships between features considered by the individual application of the feature selection. To solve this problem, in this work, we use an approach^34^ based on a graph where the nodes represent the features and the links represent the features co-occurrence in the same use of the algorithm, rather than storing the repeated application of different selectors of features as a vote vector. In this way, the method considers how many times a feature was selected and also considers the sets of features that are selected together each time the algorithm is used.

The rest of this work has been organized as follows: Section 2 describes the data set characteristics and molecular representation, the graph-based feature selection method, and the experimental setup. Section 3 describes the experimental results, and finally, Section 4 provides a summary of the conclusions of this work.

## 2 Material and Methods

In this section, we discuss the methodology used in this work, the data set, and the algorithms used.

### 2.1 Data Set Characteristics and Molecular Representation

In our study, the data were collected from different sources to yield a total of 24 data sets previously used for the construction of binary prediction models for different molecular targets. Each molecule in the data sets was represented using GSFrag,^35,36^ which considers 1138 molecular fragments (247 GSFrag + 891 GSFragl), with the fragments consisting of one or more disconnected components. Each component considers, among other factors, paths of length *n*, cycles on *m* vertices, or paths (cycles) with a number of attached chains of unit length.

In the construction of QSAR models, the diversities of the data sets play fundamental roles for the generalization of the models. Thus, models built from small or homogeneous compound sets offer poor generalization capacity.^37−39^ Recently González-Medina et al.^40^ proposed a new approach to study the diversity of molecular databases from different perspectives, including fingerprint-based diversity and the diversity of physicochemical properties. In our work, we have studied the diversity of the data sets from four perspectives: (i) fingerprint-based diversity, (ii) diversity of physicochemical properties, (iii) minority class ratio diversity, and (iv) data set size diversity (number of compounds in the data set). To evaluate the fingerprint-based diversity (FpSim), we used the Tanimoto similarity index calculated from the topological fingerprint (Morgan/Circular Fingerprints, radius = 2)^41^ for all pairs of molecules in each of the data sets. The diversity of physicochemical properties was calculated using two properties: the octanol/water partition coefficient (ALogPS) and molecular weight (MW). Fingerprint and physicochemical properties were calculated with the RDKit library.^42^

Table 1 summarizes the characteristics of the data sets. The information shown in the table includes a unique identifier for each data set, the number of total molecules, the number of active/inactive elements (positive/negative class), the coefficient of variations for ALogPS, MW, and FpSim, and a description of the molecular pathway end point. The coefficient of variation (CV) was defined as follows:
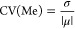
1with σ being the standard deviation, μ the mean, and Me the parameter under study (ALogPS, MW, FpSim).

**Table 1:** Data Set Characteristics

Data set	No. molecules	Class –	Class +	CV (ALogPS)	CV (MW)	CV (FpSim)/Avg (FpSim)	Description	ref
DS1	432	155	277	0.430	0.187	0.295/0.408	Inhibitors of factor Xa of the benzamidine Family	(43)
DS2	311	242	69	0.351	0.205	0.299/0.528	Inhibitors of c-Jun N-terminal Kinase-3	(38)
DS3	534	337	197	2.058	0.486	0.227/0.363	*Plasmodium falciparum* growth inhibitor assay	(44)
DS4	780	409	371	0.659	0.405	0.269/0.390	Molecules set versus *Mycobacterium tuberculosis*	(45)
DS5	1510	820	690	0.295	0.233	0.234/0.424	Inhibitors of human β secretase 1	(46), ^47^
DS6	1880	639	1241	0.494	0.283	0.235/0.366	P-glycoprotein inhibitors	(48)
DS7	483	241	242	0.740	0.474	0.259/0.383	P-glycoprotein substrates	(48)
DS8	567	260	307	0.658	0.390	0.232/0.382	Chembench: 313_MDR1	(49)
DS9	426	201	225	0.489	0.386	0.304/0.405	Chembench: 322-MRP1i10	(49)
DS10	122	61	61	1.805	0.363	0.288/0.402	Chembench: 342_MRP4x	(49)
DS11	70	35	35	0.774	0.273	0.312/0.397	Chembench: 412_NTCPx	(49)
DS12	82	41	41	2.691	0.405	0.216/0.384	Chembench: 422_OCT1x	(49)
DS13	292	139	153	1.701	0.392	0.261/0.431	Chembench: 24_PEPT1x	(49)
DS14	1219	610	609	0.679	0.350	0.212/0.361	Chembench: 151305_ebola_1224cpds_PCM4	(49)
DS15	171	64	107	0.273	0.163	0.345/0.498	Chembench: ack1	(49)
DS16	289	146	143	0.473	0.234	0.255/0.370	Chembench: BetaLactamase_Dataset_Vini	(49)
DS17	3823	1951	1872	0.256	0.181	0.214/0.374	Chembench: D2_improved_eugene	(49)
DS18	320	182	138	0.331	0.194	0.361/0.385	Chembench: IE_M1_Descriptors	(49)
DS19	369	278	91	0.970	0.628	0.375/0.310	Chembench: Ld50_impress_JRC	(49)
DS20	1919	864	1055	0.331	0.179	0.242/0.401	Chembench: IE_5-HT6_Descriptors	(49)
DS21	1290	154	1136	0.798	0.476	0.310/0.337	Estimation of aqueous solubility	(50), (51)
DS22	806	433	373	0.589	0.318	0.211/0.365	Human Ether-à-go-go-Related Gene	(51)
DS23	4054	2362	1692	0.327	0.213	0.199/0.356	Pubchem BioAssay: AID 2044	(51)
DS24	19737	19562	175	0.350	0.233	0.201/0.356	Malaria (*Plasmodium falciparum*)	(51)

In the Supporting Information, Figure S1a and b shows the distribution of physicochemical properties ALogPS and MW in each data set, while Figure S1c shows the cumulative distribution function using the pairwise similarity values of the compounds in each data set, where both representations exhibit the diversity of the data sets used. The similarity cumulative distribution function using the pairwise Tanimoto similarity with the Morgan fingerprint shows pariwise similarity values lower than 0.5 in 80% of the cases, highlighting the structural diversity of the molecules. Moreover, CV (FpSim) (Table 1) shows values equal to or less than 0.3 for a large majority of the data sets, indicating that the mean of the pairwise fingerprint (Avg (FpSim)) is representative of the data set. Considering the 24 data sets, the values of Avg (FpSim) range from 0.31 to 0.53, which confirms the structural diversity mentioned above.

In terms of molecular properties, the CV (ALogPS) and CV (MW) values show greater diversity in terms of ALogPS compared to MW. Moreover, the selected data sets present a wide range in terms of the number of compounds, with the minimum size of 70 and a maximum size of 19,737. The balance between the classes (active/inactive) also presents great diversity, with the percentage of minority classes within the range from 50% (perfectly balanced) to 0.8% (highly unbalanced).

**Figure 1:**
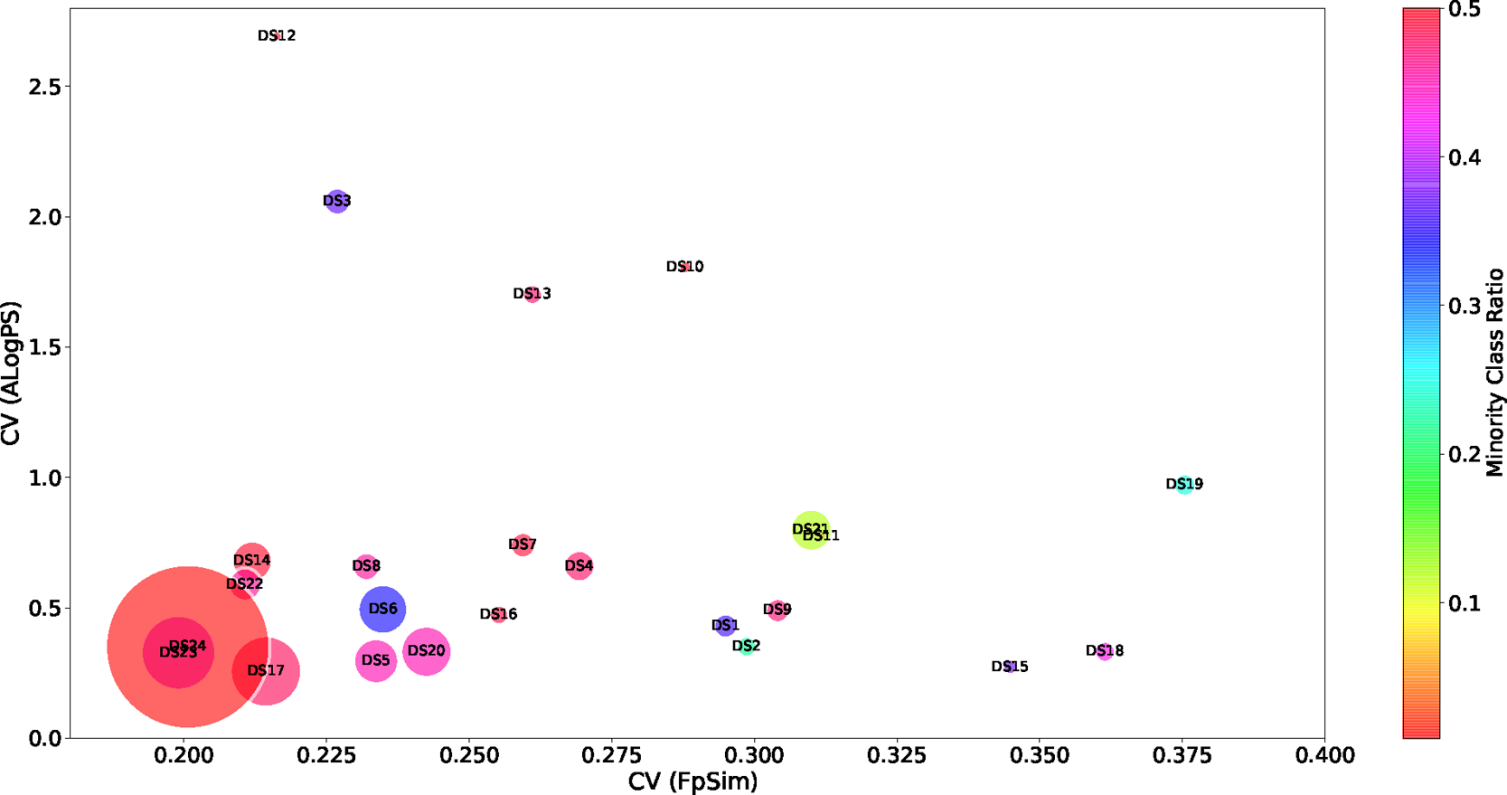
Multifactorial data set diversity representation: fingerprint (*x* axis), physicochemical properties (*y* axis), minority class ratio (color), and data set size (mark size).

Figure 1 shows a more comprehensive multifactorial representation of data set diversity, which simultaneously represents the diversity of chemical data sets by fingerprint (*x* axis), physicochemical properties (*y* axis), minority class ratio (color), and data set size (mark size).

### 2.2 Graph-Based Feature Selection Method

The feature selection ensemble approach used in this paper is based on boosting.^30^ In every feature selection boosting step, the selection is usually recorded by casting a vote for each selected feature. These votes are weighted when the corresponding boosting algorithm uses weighted classifiers as members of the ensemble. Once the Tr rounds are finished, a vector of votes is obtained that records how many times every feature has been selected, and a final selection is determined using that vector.^30^ This approach does not take into account the relationship among the features.

The algorithm utilized in this work uses a different approach based on an undirected graph in the ensemble construction.^34^ The first step of the algorithm consists of the construction of an undirected graph that allows storing the results of each feature selection process. For this, the nodes of the graph are used to store the features, and the edges represent the concurrent selection of the two features in the same application of the algorithm. In the second step, this graph is used to select a group of features from the selection, which is performed for every step of the ensemble construction process.

**Figure 2:**
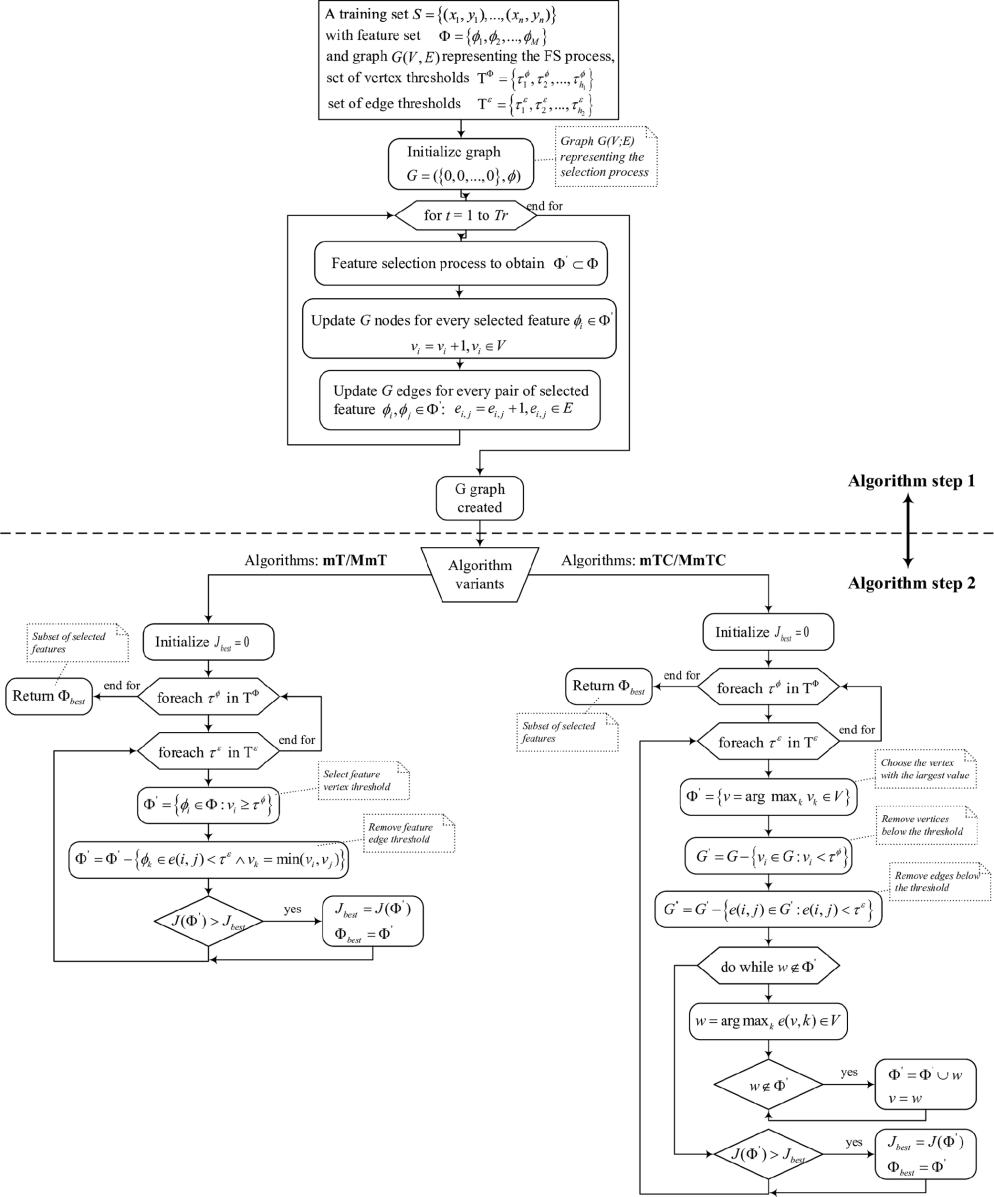
Graph-based feature selection algorithm.

Figure 2 shows the graph-based feature selection algorithm. The first step stores the feature selection results using an undirected graph *G*(*V*, *E*), representing the vertices *V* = (1, 2, ..., *M*), the features Φ = ϕ_1_, ϕ_2_, ...ϕ_*M*_, and the links representing the selection of the two features simultaneously. For simplicity, we consider that the vertex *i* coincides with the feature ϕ_*i*_ and is assigned the value *v*_*i*_. Moreover, a member (*i*, *j*) of the set *E* corresponds to a link between *i* and *j* with an *e*(*i*, *j*) value.

Once a feature selection method is applied in the ensemble construction (FAST method^8^ in this work), the vertices corresponding to the selected features and the edges linking these vertices are increased by one. If the boosting method is used for feature selection,^30,52^ the votes used for the *t*th member of the ensemble can be weighted by a value α_*t*_; in this way, the value is increased by α_*t*_ instead of increasing by one. Finally, the vertex values in the created graph reflect how many times each feature was selected and the link values how many times two features were selected together.

The method is based on the assumption that the features that represent the nonredundant information are those that are most frequently selected together and not those that rarely occur. The second step of the algorithm uses the created graph to select a set of features; for this purpose, it is necessary to establish two thresholds: the first for the vertices (τ^ϕ^) and the second for the edges (τ^ϵ^). Using these thresholds, it is possible to select a set of features Φ′(τ^ϕ^, τ^ϵ^) by applying different strategies.

To select a subset of Φ′(τ^ϕ^, τ^ϵ^) features, these two terms were evaluated using reduction, *r*(Φ′(τ^ϕ^, τ^ϵ^)), and the performance in classification, measured by Cohen’s κ value, κ(Φ′(τ^ϕ^, τ^ϵ^)). Thus, considering *m* selected features from all *M* features set, the reduction was measured as , and both metrics were combined using the following equation
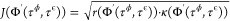
2

The highest performance, *J*, is chosen by evaluating all possible vertex and edge threshold pairs. As possible thresholds, all the different values for vertices and edges or a fixed number can be considered, and in this case, the values can be divided into equal subintervals. Once the values for a pair (τ^ϕ^, τ^ϵ^) have been set, the feature selection process proceeds in the following way: initially, for the vertex *v*_*i*_ with a value greater than or equal to τ^ϕ^, the features ϕ_*i*_ are selected. Then, for every pair of selected features (ϕ_*i*_, ϕ_*j*_), if the corresponding edge, *e*(*i*, *j*), is below the edge threshold, *e*(*i*, *j*) < τ^ϵ^, feature ϕ_*i*_ is removed if *v*_*i*_ < *v*_*j*_ or feature ϕ_*j*_ is removed otherwise. With a vertex threshold of 0, the method includes the particular case of a standard voting scheme in which all voting combinations are considered.

Using this first strategy, two variants of the algorithms are considered: (i) the mT variant, where τ^ϕ^ = 0 and τ^ϵ^ ≠ 0, and (ii) the MmT variant, where τ^ϕ^ ≠ 0 and τ^ϵ^ ≠ 0. These two methods are shown in the left-hand side of the algorithm shown in Figure 2.

The second strategy of the algorithm (right-hand side in step 2 of the algorithm, Figure 2) is based on forming a chain of features using the following procedure. First, the feature corresponding to the vertex with the largest value is selected. Then, for these vertex edges, the largest value above the given threshold τ^ϵ^ is chosen, and the corresponding feature is added to the set of selected features. In this way, this feature becomes the new starting point, and the process ends when all of the edges above the threshold from the current last member of the chain are linked to already-selected vertices. In this second strategy, two variants were also considered: mTC, for τ^ϕ^ = 0 and τ^ϵ^ ≠ 0, and MmTC, for τ^ϕ^ ≠ 0 and τ^ϵ^ ≠ 0.

### 2.3 Experimental Setup

The experiments were performed following the protocol shown in Figure S2 of the Supporting Information. Each data set was divided randomly into two disjoint sets: one to build the model and the other to perform the external validation. Thus, following a repeated double cross-validation procedure,^53,54^ five external validation rounds (external loop in Figure S2) were completed. The feature selection process was conducted using the subset of molecules that were used to build the model.

The graph-based method was evaluated with three different well-known classifiers, a decision tree (DT), a support vector machine (SVM), and a random forest (RF). To set the hyperparameters of the classifiers in the model’s construction (inner loop in Figure S2), we used a 10-fold internal cross-validation process.

For each classifier, we identified the best hyperparameter values from a set of possible values. For RF, we used a size of 100 trees and the Gini impurity criterion to measure the quality of a split. The nodes were expanded until all leaves were pure or until all leaves contained less than two samples, and bootstrap samples were used for building the trees. For SVMs, three parameters were set: the kernel type, the *C* value, and for the Gaussian kernel, the γ value. Thus, we tested a linear kernel with *C* ∈ {0.1, 1, 10} and a Gaussian kernel with *C* ∈ {0.1, 1, 10} and γ ∈ {0.0001, 0.001, 0.01, 0.1, 1, 10}. All 21 possible combinations were evaluated. For decision trees, we used 1 and 10 trials with the option of softening the thresholds and tested all four possible combinations.

The performance achieved by a classifier was measured using the geometric mean (G-Mean) of sensitivity and specificity,^55^ defined as
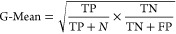
3where TP, TN, FP, and FN are the true positives, true negatives, false positives, and false negatives, respectively. The use of this metric is recommended for both balanced and unbalanced data sets because it takes into account the uneven distribution of class instances.

The reduction capacity for feature selection methods can be defined as follows
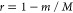
4where *m* is the number of selected features, and *M* denotes the total number of features.

Redundancy was evaluated using two different metrics: one based on mutual information and the second based on the correlation.^56^ Mutual information can be defined as follows
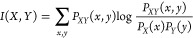
5where *X* and *Y* depict feature vector and class vector, respectively, and *P*(·) represents probability.

Consider *S* to be a vector of a given set of features and *h* a class variable. The redundancy based on mutual information (*MI*) was measured as
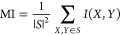
6where |*S*| is the number of features in *S*.

Redundancy based on correlation (AcRed) was evaluated by replacing mutual information with a correlation coefficient.^56^ For this purpose, the mean (meanC) of the correlation coefficient was calculated. Thus, AcRed was defined as follows
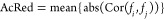
7where *f*_*i*_, *f*_*j*_ ∈ *S∀i*, *j* = 1, 2, ..., *m*, and Cor() is a correlation coefficient function and abs() an absolute value function.

To guarantee a rigorous comparison between the graph-based feature selection method and the standard method, it is necessary to use specially designed statistical tests to evaluate multiple algorithms on multiple data sets. To do this, it is first necessary to know whether there is a significant difference among the methods. The Iman–Davenport test is recommended to determine the existence of these statistical differences. It is based on the χ_*F*_^2^ Friedman test, which compares the average ranks (*R*) of *k* algorithms for *N* data sets, but it is more powerful.^57^

After applying the Iman–Davenport test, it is necessary to apply some of the general procedures for controlling the family-wise error in multiple testing. The Holm test^57^ is designed to compare in a stepwise manner the algorithm with the best performance in terms of Friedman ranges with the rest of the methods under study. The statistical test for comparing the *i*th and *j*th methods is defined as follows
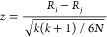
8

Using the normal distribution table, the values of *z* were used to find the corresponding probability. Step-down procedures sequentially test the hypotheses in order of their significance; the ordered *p* values were denoted by *p*_1_, *p*_2_..., such that *p*_1_ ≤ *p*_2_... ≤ *p*_*k*–1_. The Holm method compares each *p*_*i*_ with α/(*k* – 1). The step-down procedure starts with the most significant *p* value. If *p*_1_ is below α/(*k* – 1), the corresponding hypothesis is rejected, and we compare *p*_2_ with α/(*k* – 1). If the second hypothesis is rejected, the test proceeds to the third and so on. When a null hypothesis cannot be rejected, all remaining hypotheses are retained as well. For all tests, we used a significance level α = 0.05.

To compare multiple algorithms, we used the Nemenyi^58^ test. This test considers the performances of two algorithms to be significantly different if the corresponding average Friedman’s ranks differ by at least the critical difference. The critical difference (CD) for *N* data sets and *k* algorithms was formulated as follows^57,58^
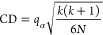
9

## 3 Experimental Results

In order to assess the effectiveness of the proposed feature selection approach in the construction of binary QSAR models for molecular activity prediction, we performed different experiments with the three classifiers mentioned above with respect to 24 data sets with different molecular activities. As stated, the algorithm supports the use of different feature selection methods. We used fast clustering-based feature selection (FAST) in the tests.^8^ The standard voting AdaBoost method (T) was used as a reference to make the comparisons. The experimental results are included in Tables S1–S3 of the Supporting Information.

**Figure 3:**
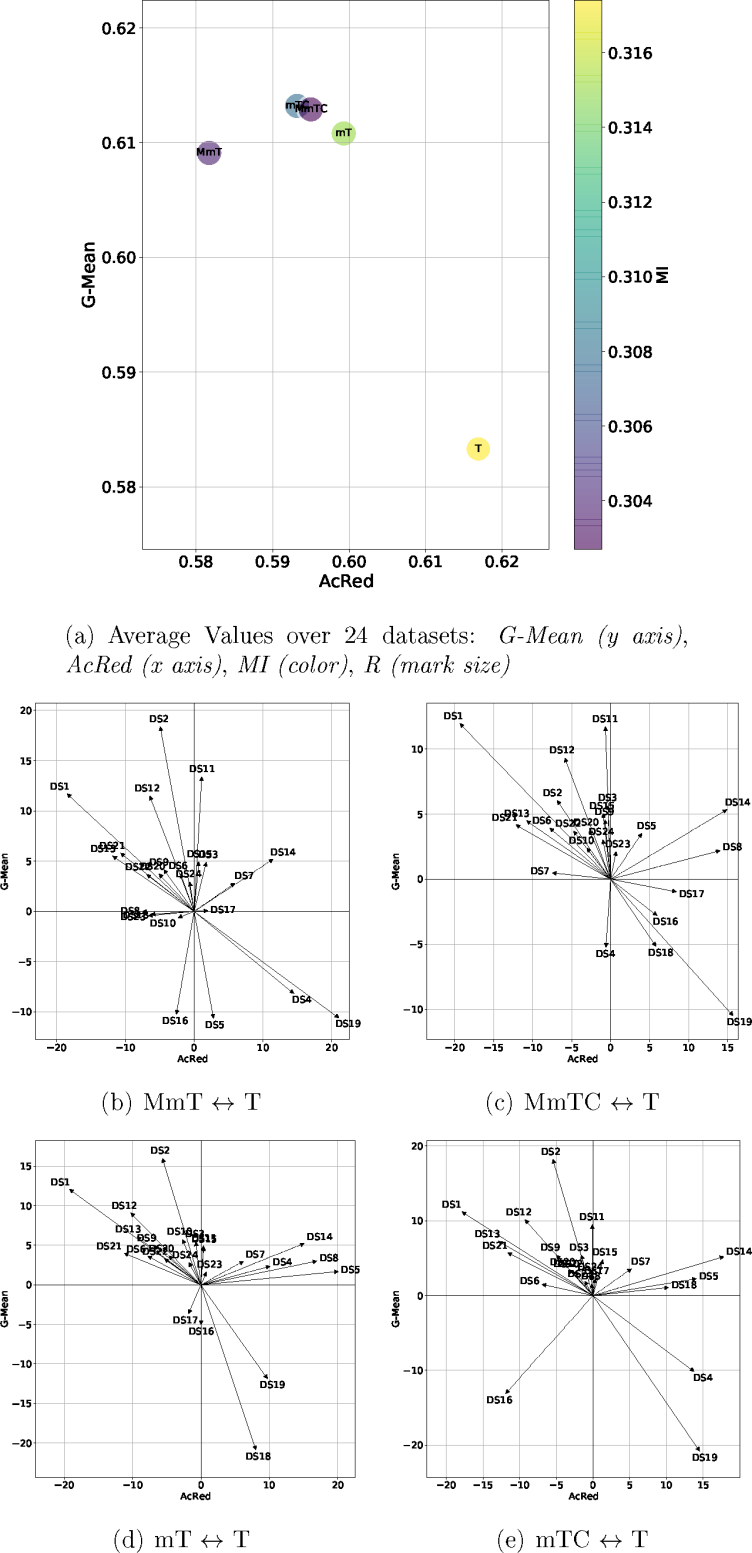
Four metrics results (a) and the moment diagrams (b–e) representing the differences (G-Mean) of the proposals against the base method (T) for the DT classifier.

**Figure 4:**
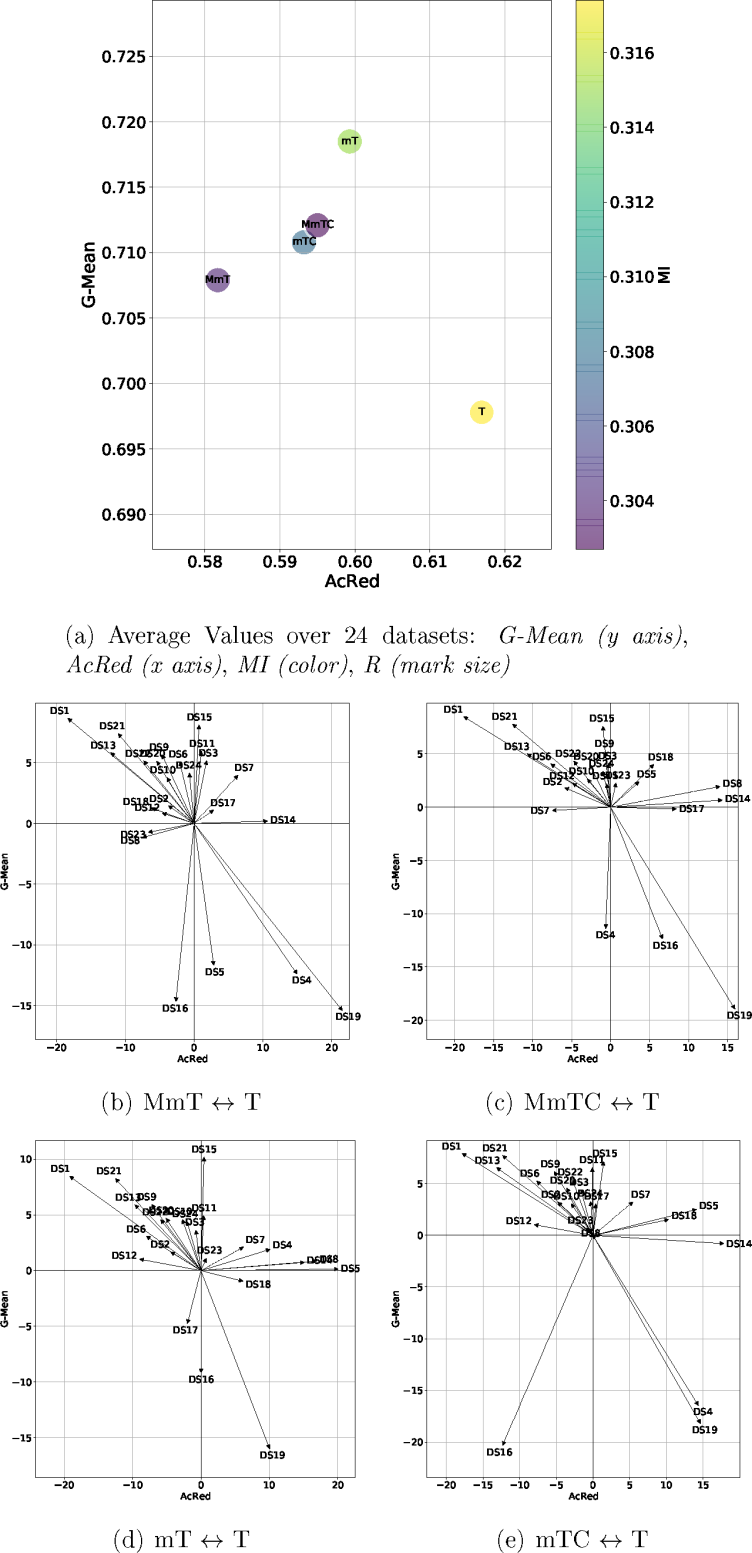
Four metrics results (a) and the moment diagrams (b–e) representing the differences (G-Mean) of the proposals against the base method (T)) for the RF classifier.

**Figure 5:**
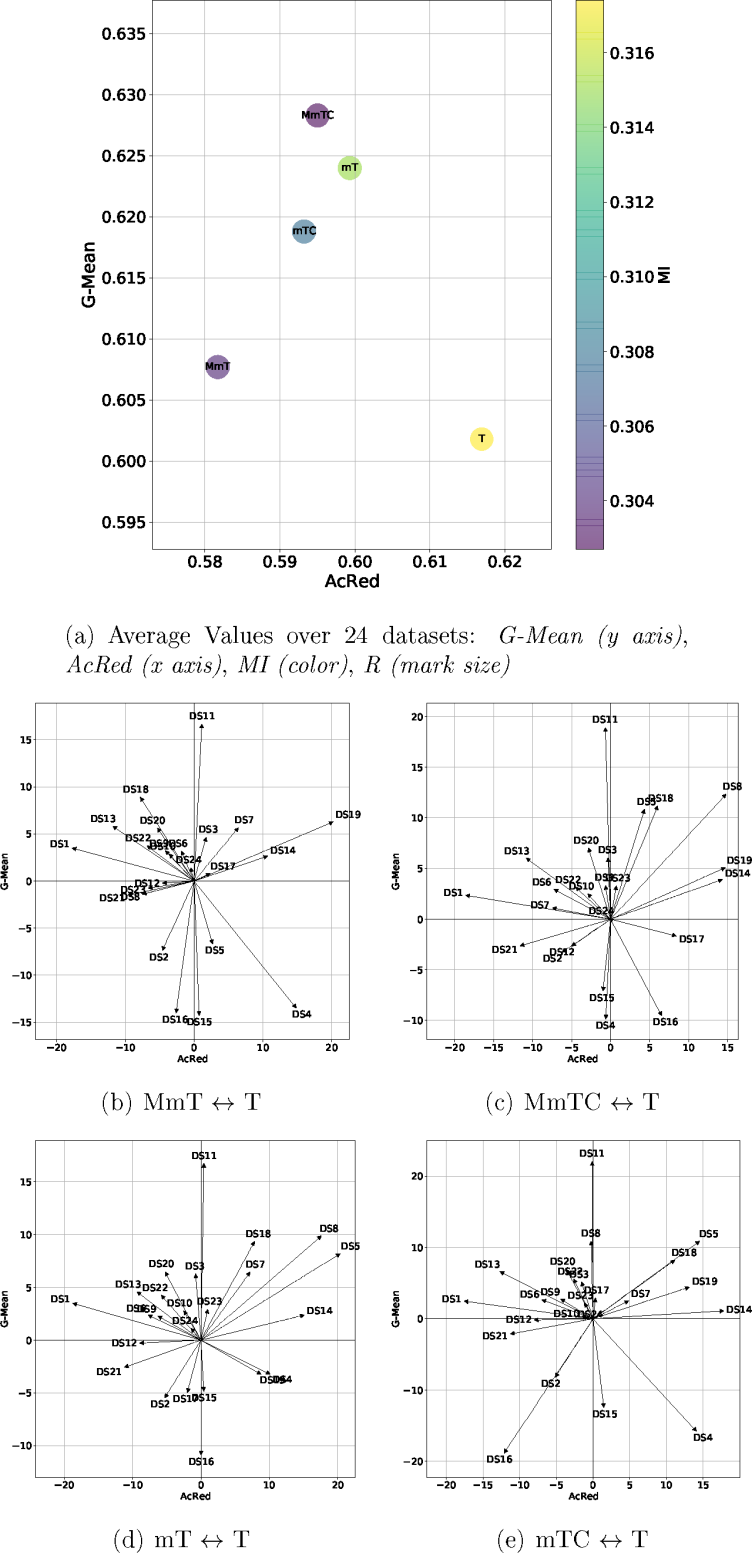
Four metrics results (a) and the moment diagrams (b–e) representing the differences (G-Mean) of the proposals against the base method (T) for the SVM classifier.

In this section, we present a graphical representation (Figures 3–5) of the results for ease of presentation and discussion. These figures include two types of representation: the first (panel (a) in Figures 3–5) represents the average values of four metrics G-Mean (*y* axis), AcRed (x axis), MI (color), and R (mark size), while the second (panels (b)–(e) in Figures 3–5) represents the performance of two metrics (G-Mean, AcRed) for each data set.^59^ To construct this 2D representation, the value of each axis represents the difference of the graph-based methods (MmT, MmTC, mT, mTC) with respect to the base method (T) for the same data set. In this way, the arrows pointing downward-left represent the data sets for which the base T algorithm outperformed our graph-based method for G-Mean and was worse in terms of AcRed, the arrows pointing upward-left indicate that our graph-based method improved the G-Mean and AcRed. The arrows pointing upward-right show data sets for which our graph-based method improved the G-Mean but had an inferior AcRed, and arrows pointing downward-right show the data sets for which the base T algorithm outperformed our graph-based method with respect to both G-Mean and AcRed. In the figures, the values of the differences are represented as percentages.

Figure 3 shows the results for the DT classifier. The results in terms of G-Mean (Figure 3a) showed a better performance for the graph-based methods compared to the base method T. This better behavior in terms of G-Mean was also shown by the number of data sets for which the use of some of the graph-based methods outperformed T. For example, with the application of MmT (Figure 3b), G-Mean was improved in 17 of the 24 data sets evaluated. MmTC (Figure 3c) improved it in 19 of them. mT (Figure 3d) improved it in 20 of them, and mTC (Figure 3e) improved it in 21 of them.

Regarding redundancy, the mean values obtained for MI and AcRed also showed better results (lower values) for the graph-based method compared to the base method (*x* axis and color scale values in Figure 3a). According to the distribution of the differences for each data set in terms of AcRed, the best result was obtained with the application of the methods MmTC, mT, and mTC, where 16 of the 24 data sets obtained better results compared to the base method T. Moreover, for reduction (R), the results were very similar for all of the methods.

Figure 4 shows the results for the RF classifier. The overall performance for RF in terms of G-Mean was better than that using DTs. Higher average values were obtained, and the advantage of using the graph-based methods with respect to the base method T was retained. In terms of redundancy (MI, AcRed), values very similar to those achieved by DT were obtained, with better performance for graph-based methods regarding T. For RF, the distribution by data set achieved the best results for the mT and mTC methods, which have surpassed the T method by 20 data sets in terms of G-Mean and by 18 data sets in terms of AcRed.

For the SVM classifier (Figure 5), although the values of G-Mean were lower than RF, they showed similar global behavior to those obtained by both DT and RF, showing that the G-Mean had a better performance with the use of MmT, MmTC, mT, and mTC compared to the application of T. The values of R, MI, and AcRed also behaved in a similar way to the results presented for the DT and RF classifiers, observing a decrease in redundancy (MI, AcRed) when the MmT, MmTC, mT, and mTC methods were applied.

Although the results presented so far show the benefits of using the graph-based method compared to the standard method, to obtain conclusive results, the benefits must be validated by means of statistical tests. First, we tested global significant differences using Iman–Davenport. If this test rejected the null hypothesis, we used the Holm procedure to compare the best method with the rest of the methods and then the Nemenyi test to perform a global comparison of all the methods.

**Figure 6:**
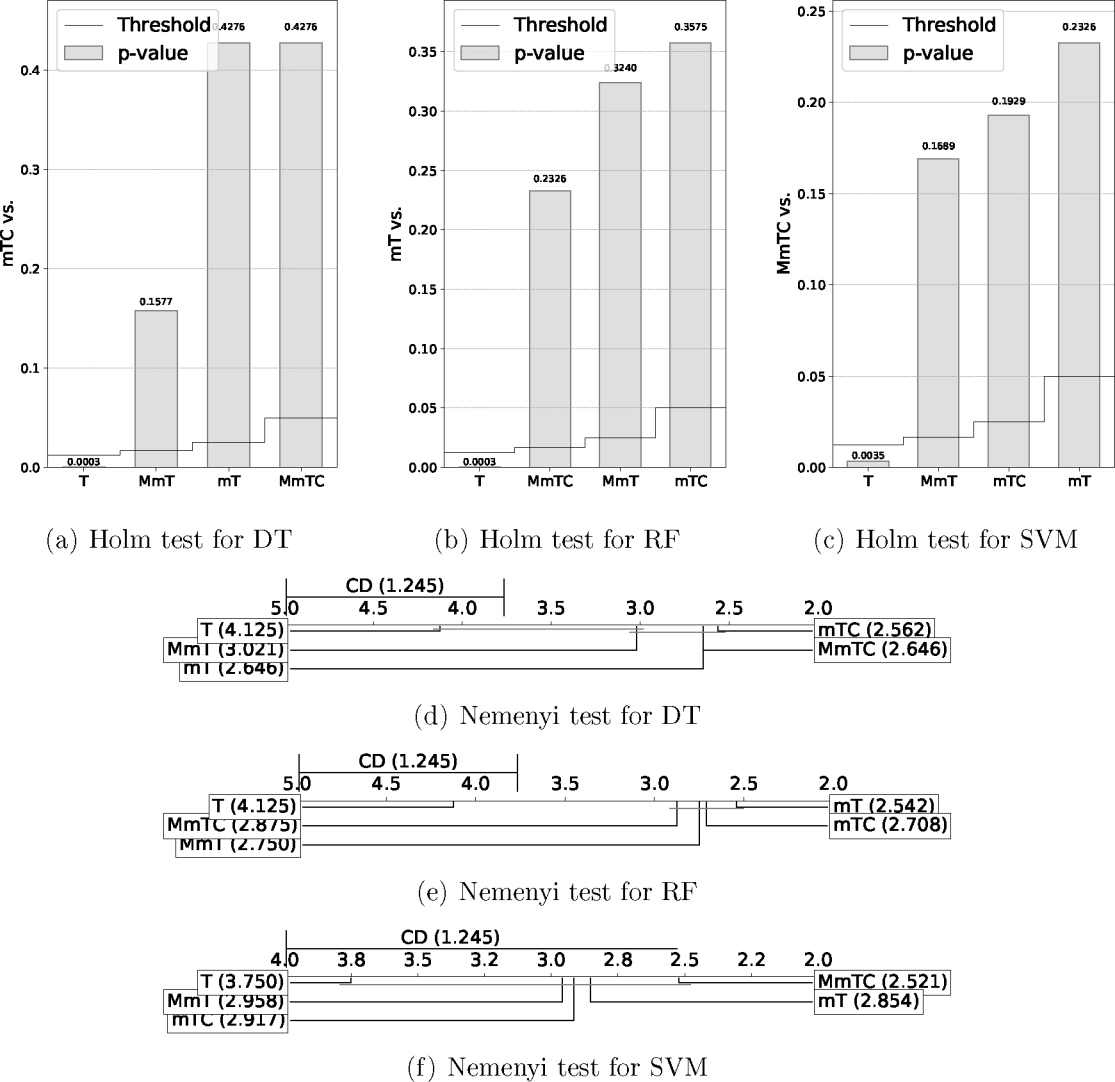
Performance statistical test results representation in terms of G-Mean.

**Figure 7:**
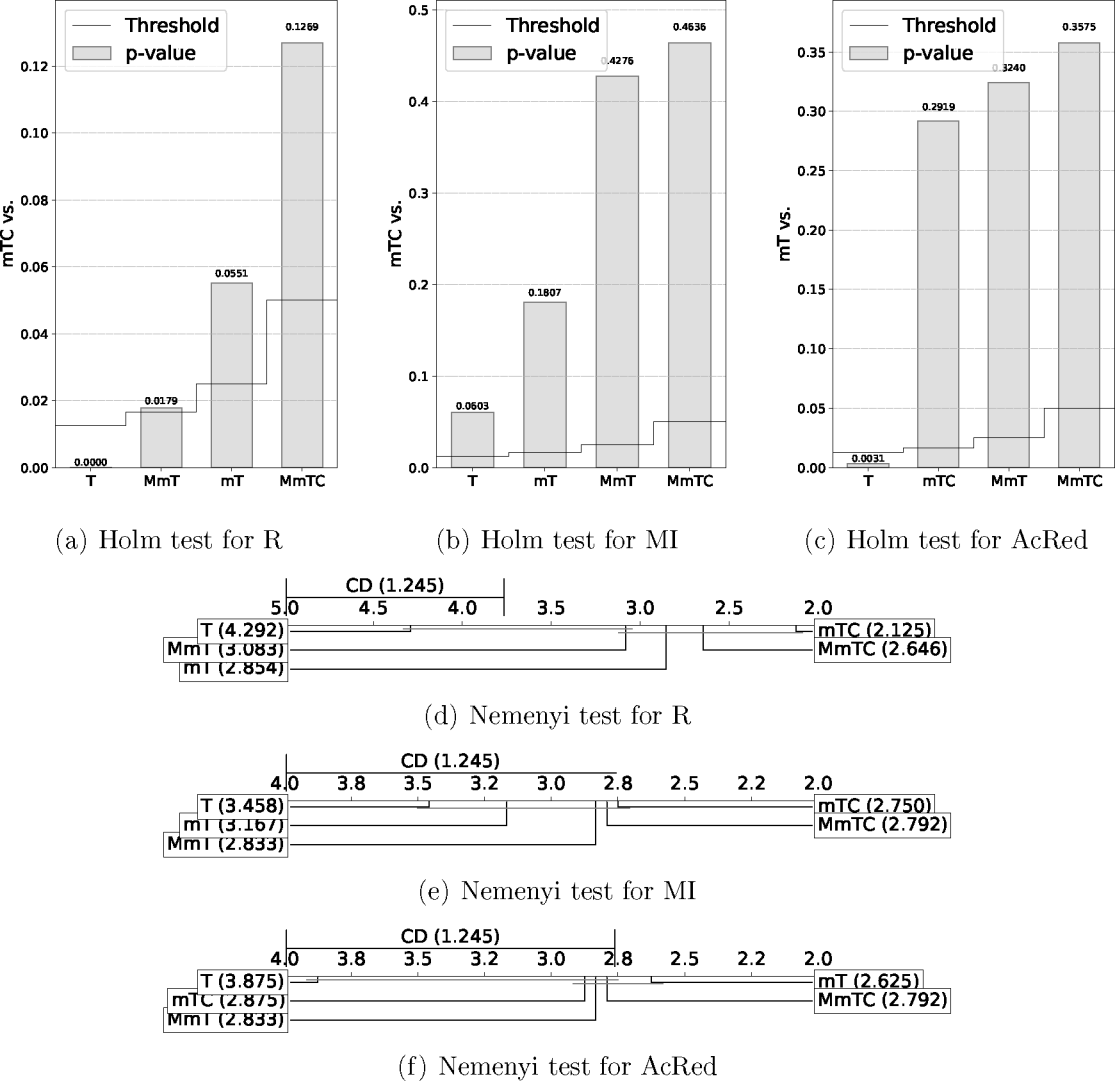
Reduction and redundancy statistical test results representation.

Tables 2 and 3 show the results of these statistical tests, and Figures 6 and 7 show a graphical representation of these results in order to facilitate comparisons.^57^ Holm graphs show the best of the algorithms on the *y* axis and use a bar graph to represent the *p* values and a line graph to represent the thresholds. The Nemenyi graphs connect with a horizontal line the groups of algorithms that were not significantly different and show the critical difference in the upper left corner of the graph.

**Table 2:**
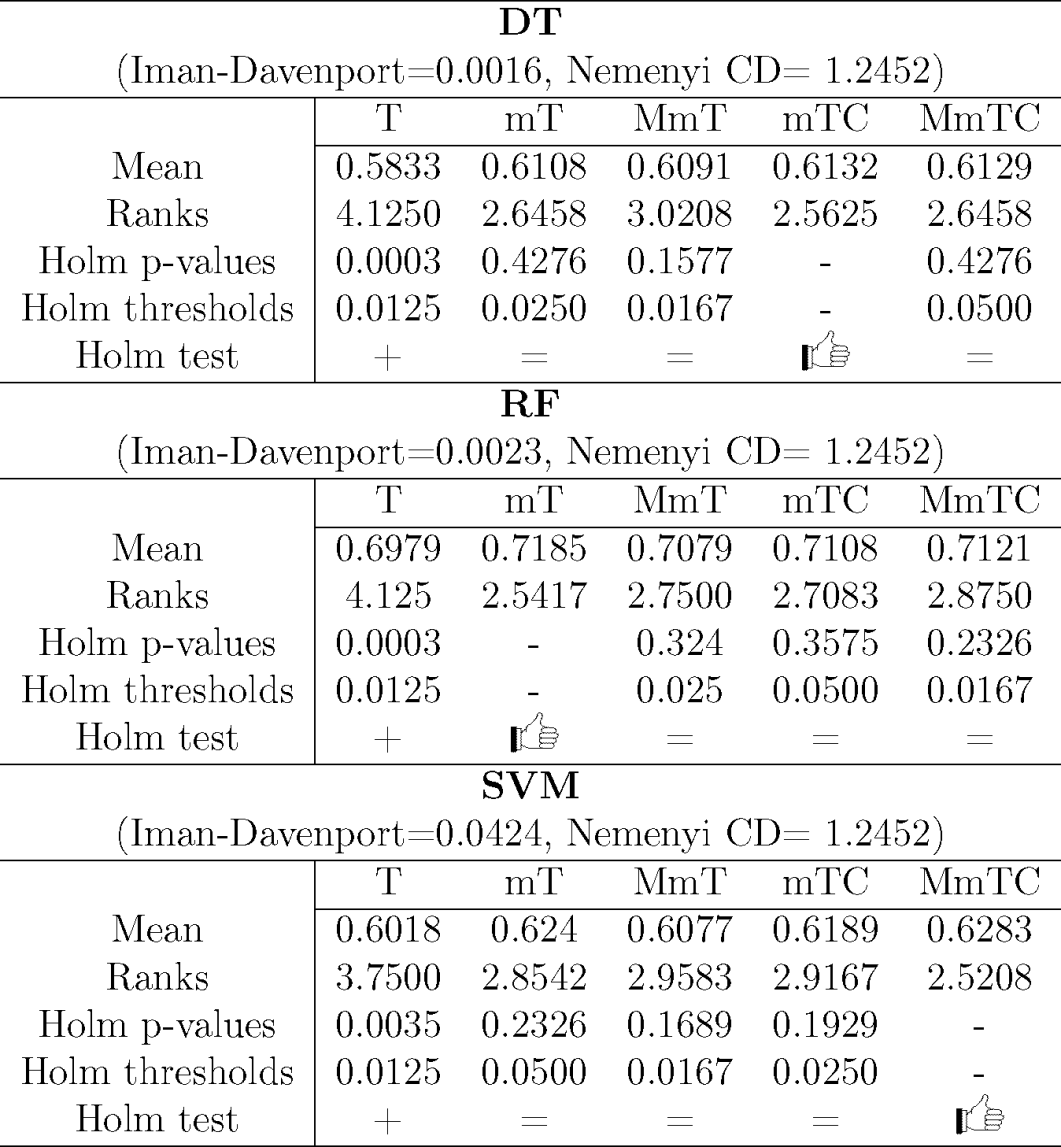
Performance (G-Mean) Statistical Tests Results^a^

a The best method according to the Holm test is indicated by the thumbs up hand symbol.

**Table 3:**
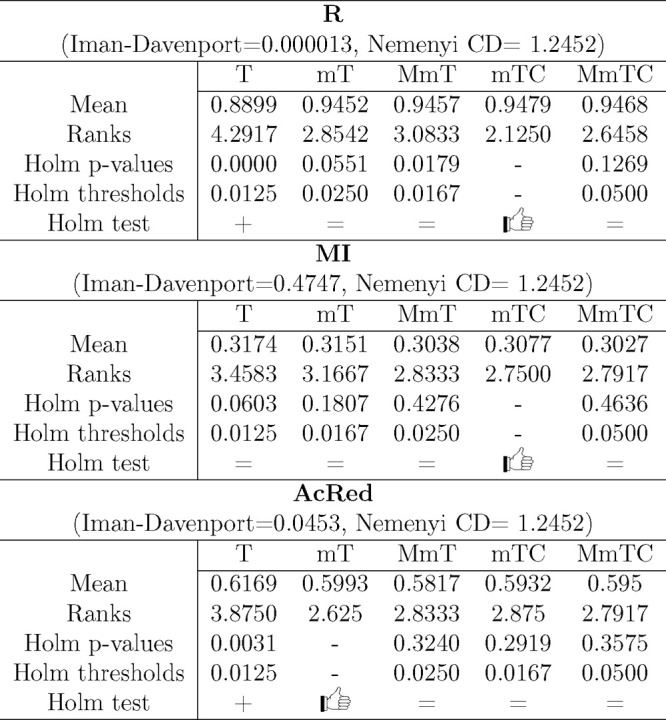
Reduction and Redundancy Statistical Tests Results for RF^a^

a The best method according to the Holm test is indicated by the thumbs up hand symbol.

As shown in Table 2 and Figure 6 for the performance of classifiers in terms of G-Mean, the result obtained for the Iman–Davenport test was very close to zero, demonstrating the existence of a significant difference between the evaluated methods. Moreover, as shown in Figure 6, for all classifiers, the methods selected as the best by Holm test showed significant differences with respect to the base method (T), with no significant differences shown with respect to the rest of the compared methods. The methods selected as the best were the following: mTC for DT, mT for RF, and MmTC for SVM.

The Nemenyi test confirmed these results, with the base method (T) performing worst for all classifiers. However a different behavior was observed for DT as the differences were not significant between MmT and T, indicating significant differences of mTC and MmTC with T. RF showed significant differences for all of the methods with respect to T, and SVM did not achieve significant differences among all the compared methods.

As shown in Table 3 and Figure 7, in terms of reduction (R), the results of the Iman–Davenport test showed significant differences. For redundancy, a significant difference was obtained for AcRed but not for MI.

The best method in terms of Friedman’s ranks for R and MI was the mTC method, and the best in terms of AcRed was mT. In all cases, the base method T produces the worst results, with a significant difference (below the threshold) in terms of R and AcRed.

The results of the Nemenyi test confirmed the worst results obtained by T in terms of R, MI, and AcRed. Moreover, in terms of R, the results did not show a significant difference between the T and MmT methods, with the best performances observed for the mTC and MmTC methods with significant differences compared to T. In terms of AcRed, the best performance was obtained for the mT method, with significant differences with respect to T, and in terms of MI, the results did not show a significant difference.

**Figure 8:**
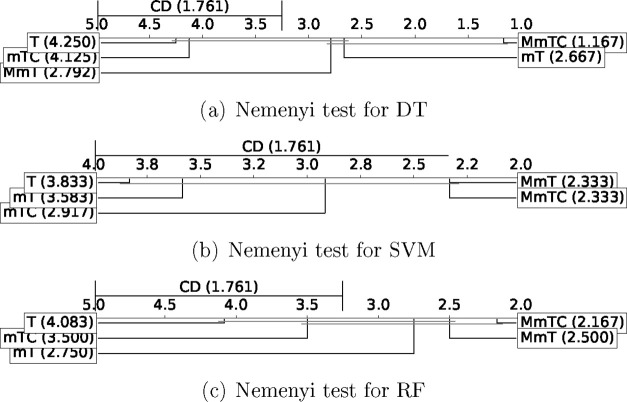
Performance Nemenyi test results for TOX-21 benchmark in terms of G-Mean.

**Figure 9:**
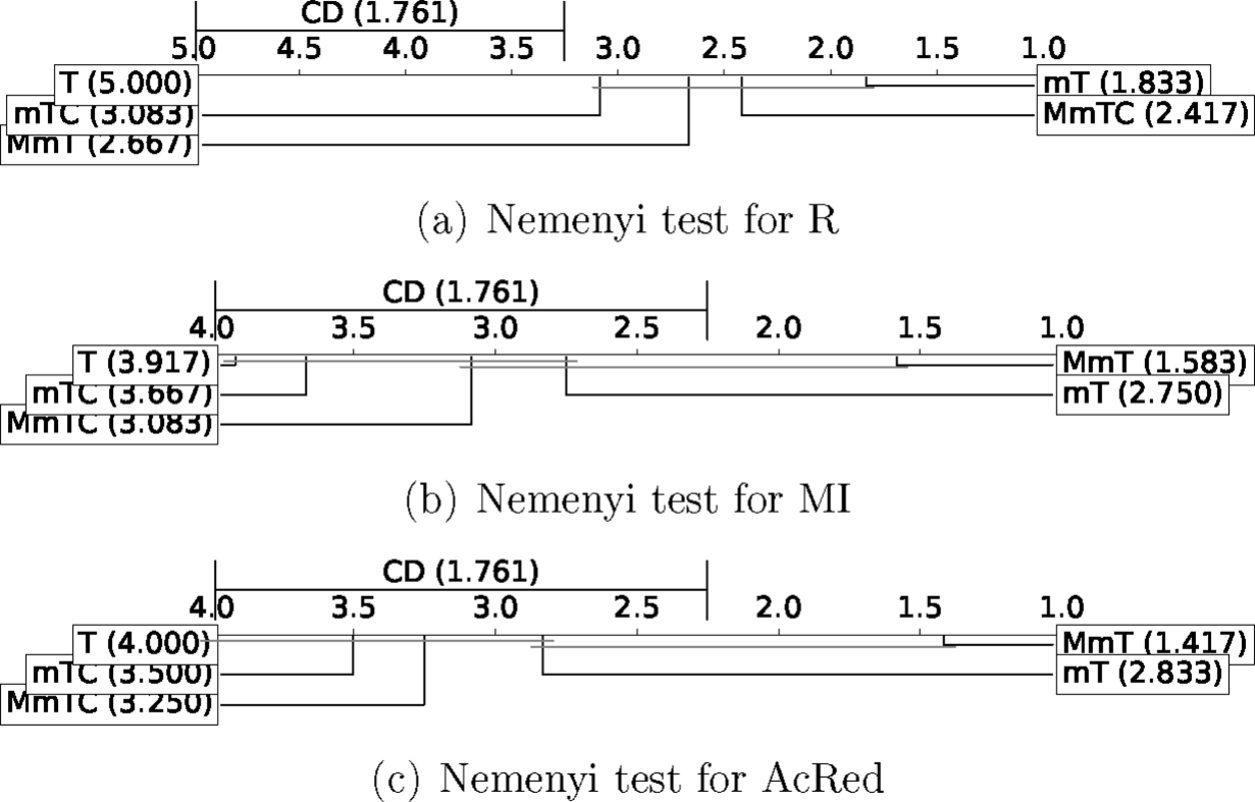
Reduction and redundancy Nemenyi test results for TOX-21 benchmark.

Finally, the experiments were extended evaluating their application to the prediction of toxicity. For this purpose, the benchmark proposed in the Tox21 project^60−62^ was used. Figures 8 and 9 show the results of the Nemenyi test in terms of performance and of reduction and redundancy, respectively. The experimental results are included in Tables S5–S9 of the Supporting Information.

In terms of G-Mean (Figure 8), the best results for the DT and RF classifiers were observed for the MmTC, MmT, and mT methods, obtaining significant differences for these methods with respect to the base method T. For the case of the SVM classifier, the best results were obtained for MmTC and MmT methods, with significant differences with the base method T.

As Figure 9 shows, in terms of reduction (R), the proposals outperform the base method. In terms of redundancy, the proposals outperform the base method in terms of MI and AcRed. The best results were obtained for the MmT and mT methods.

## 4 Conclusions

In this work, we evaluated the application to the prediction of molecular activity of a new feature selection approach, based on the construction of an undirected graph to combine the base application selectors to different features sets. In contrast to the standard voting approach, the method not only considers the frequency which with a feature is selected but also the relationship with other features. Compared with the use of the standard voting method (T), the experimental results for different scenarios that include the DT, RF, and SVM classifiers showed the advantages of the graph-based methods mTC, MmTC, mT, and MmT in terms of classifier performance. Among the graph-based methods, the mTC method showed a better overall performance. One of the main advantages of the graph-based method is that any standard feature selection algorithm can be applied, thus opening new lines of research. Furthermore, the same idea could be adapted to the instance selection problem or the joint selection of features and instances for the construction of QSAR models.

## Data and Software Availability

All the data on which the conclusions of the work are based have been exhaustively presented in the manuscript. Data sets DS1–DS24 used in the paper are included as Supporting Information. The toxicity data sets can be download from the following Tox21 project link: https://tripod.nih.gov/tox21/challenge/. The source code under GNU General Public License v3.0 can be downloaded from the following link: http://cib.uco.es/wp-content/uploads/2021/11/source.zip

## Supporting Information Available

Experimental results in terms of R, MI, AcRed, G-Mean for each data set (Tables S1–S9); distribution of physicochemical properties ALogPS and MW in each data set (Figure S1a, b); cumulative distribution function using the pairwise similarity values (Figure S1c); and experimental setup (Figure S2)(PDF)
SDF files with the molecular representation and activity (class) values for data sets DS1–DS24 (ZIP)

## References

[ref1] Masoudi-Sobhanzadeh Y.; Motieghader H.; Masoudi-Nejad A. FeatureSelect: a software for feature selection based on machine learning approaches. BMC Bioinformatics 2019, 20, 170 10.1186/s12859-019-2754-0. 30943889 PMC6446290

[ref2] Eklund M.; Norinder U.; Boyer S.; Carlsson L. Choosing Feature Selection and Learning Algorithms in QSAR. Journal of Chemical Information and Modeling 2014, 54, 837–843. 10.1021/ci400573c. 24460242

[ref3] Hoque N.; Bhattacharyya D. K.; Kalita J. K. MIFS-ND: A mutual information-based feature selection method. Expert Systems with Applications 2014, 41, 6371–6385. 10.1016/j.eswa.2014.04.019.

[ref4] Witten I. H.; Frank E.; Hall M. A.; Pal C. J. Data Mining: Practical Machine Learning Tools and Techniques; Morgan Kaufmann, 2016.

[ref5] Tang J.; Alelyani S.; Liu H. Feature selection for classification: A review. In Data Classification: Algorithms and Applications; Taylor & Francis Group, 2014.

[ref6] Yu L.; Liu H. Feature selection for high-dimensional data: A fast correlation-based filter solution. Proceedings of the 20th International Conference on Machine Learning (ICML-03), 2003; pp 856–863.

[ref7] Weston J.; Pérez-Cruz F.; Bousquet O.; Chapelle O.; Elisseeff A.; Schölkopf B. Feature selection and transduction for prediction of molecular bioactivity for drug design. Bioinformatics 2003, 19, 764–771. 10.1093/bioinformatics/btg054. 12691989

[ref8] Song Q.; Ni J.; Wang G. A fast clustering-based feature subset selection algorithm for high-dimensional data. IEEE Trans. Knowl. Data Eng. 2013, 25, 1–14. 10.1109/TKDE.2011.181.

[ref9] Robnik-Šikonja M.; Kononenko I. Theoretical and empirical analysis of ReliefF and RReliefF. Machine learning 2003, 53, 23–69. 10.1023/A:1025667309714.

[ref10] Onay A.; Onay M.; Abul O. Classification of nervous system withdrawn and approved drugs with ToxPrint features via machine learning strategies. Computer methods and programs in biomedicine 2017, 142, 9–19. 10.1016/j.cmpb.2017.02.004. 28325450

[ref11] Tung C.-W. Acquiring decision rules for predicting ames-negative hepatocarcinogens using chemical-chemical interactions. IAPR International Conference on Pattern Recognition in Bioinformatics, 2014; pp 1–9.

[ref12] Guo G.; Neagu D.; Cronin M. T. A study on feature selection for toxicity prediction. International Conference on Fuzzy Systems and Knowledge Discovery, 2005; pp 31–34.

[ref13] Heikamp K.; Bajorath J. How do 2D fingerprints detect structurally diverse active compounds? Revealing compound subset-specific fingerprint features through systematic selection. Journal of chemical information and modeling 2011, 51, 2254–2265. 10.1021/ci200275m. 21793563

[ref14] Ancuceanu R.; Dinu M.; Neaga I.; Laszlo F. G.; Boda D. Development of QSAR machine learning-based models to forecast the effect of substances on malignant melanoma cells. Oncol. Lett. 2019, 17, 4188–4196. 10.3892/ol.2019.10068. 31007759 PMC6466999

[ref15] Sun G.; Fan T.; Sun X.; Hao Y.; Cui X.; Zhao L.; Ren T.; Zhou Y.; Zhong R.; Peng Y. In silico prediction of O6-methylguanine-DNA methyltransferase inhibitory potency of base analogs with QSAR and machine learning methods. Molecules 2018, 23, 2892 10.3390/molecules23112892. PMC6278368 30404161

[ref16] Xiaolong D.; Siqiao T.; Yuan C.; Zheming Y. QSAR Study on the toxicities of alcohols and phenols based on minimal redundancy maximal relevance and distance correlation feature selection methods. Res. J. Biotechnol. 2016, 11, 1–6.

[ref17] Lu J.; Zhang P.; Bi Y.; Luo X. Analysis of a drug target-based classification system using molecular descriptors. Combinatorial chemistry & high throughput screening 2016, 19, 129–135. 10.2174/1386207319666151110122335. 26552442

[ref18] Hasanloei M. A. V.; Sheikhpour R.; Sarram M. A.; Sheikhpour E.; Sharifi H. A combined Fisher and Laplacian score for feature selection in QSAR based drug design using compounds with known and unknown activities. J. Comput.-Aided Mol. Des. 2018, 32, 375–384. 10.1007/s10822-017-0094-6. 29280033

[ref19] Demel M. A.; Janecek A. G.; Thai K.-M.; Ecker G. F.; Gansterer W. N. Predictive QSAR models for polyspecific drug targets: The importance of feature selection. Current Computer-Aided Drug Design 2008, 4, 91–110. 10.2174/157340908784533256.

[ref20] Hemmateenejad B.; Mehdipour A.; Deeb O.; Sanchooli M.; Miri R. Toward an Optimal Approach for Variable Selection in Counter-Propagation Neural Networks: Modeling Protein-Tyrosine Kinase Inhibitory of Flavanoids Using Substituent Electronic Descriptors. Molecular informatics 2011, 30, 939–949. 10.1002/minf.201100081. 27468149

[ref21] Zhang C.; Cheng F.; Sun L.; Zhuang S.; Li W.; Liu G.; Lee P. W.; Tang Y. In silico prediction of chemical toxicity on avian species using chemical category approaches. Chemosphere 2015, 122, 280–287. 10.1016/j.chemosphere.2014.12.001. 25532772

[ref22] Wacker S.; Noskov S. Y. Performance of machine learning algorithms for qualitative and quantitative prediction drug blockade of hERG1 channel. Computational Toxicology 2018, 6, 55–63. 10.1016/j.comtox.2017.05.001. 29806042 PMC5967266

[ref23] Martínez-López Y.; Barigye S. J.; Martínez-Santiago O.; Marrero-Ponce Y.; Green J.; Castillo-Garit J. A. Prediction of aquatic toxicity of benzene derivatives using molecular descriptor from atomic weighted vectors. Environmental toxicology and pharmacology 2017, 56, 314–321. 10.1016/j.etap.2017.10.006. 29091819

[ref24] Cardoso Gajo G.; Rodrigues Silva D.; Barigye S. J.; da Cunha E. F. F. Multi-objective Optimization of Benzamide Derivatives as Rho Kinase Inhibitors. Molecular informatics 2018, 37, 1700080 10.1002/minf.201700080. 28876533

[ref25] Bharti D. R.; Hemrom A. J.; Lynn A. M. GCAC: galaxy workflow system for predictive model building for virtual screening. BMC Bioinf. 2019, 19, 199–206. 10.1186/s12859-018-2492-8. PMC7394323 30717669

[ref26] Kharangarh S.; Sandhu H.; Tangadpalliwar S.; Garg P. Predicting inhibitors for multidrug resistance associated protein-2 transporter by machine learning approach. Combinatorial chemistry & high throughput screening 2018, 21, 557–566. 10.2174/1386207321666181024104822. 30360705

[ref27] Schöning V.; Krähenbühl S.; Drewe J. The hepatotoxic potential of protein kinase inhibitors predicted with Random Forest and Artificial Neural Networks. Toxicology letters 2018, 299, 145–148. 10.1016/j.toxlet.2018.10.009. 30315951

[ref28] Shen W.; Xiao T.; Chen S.; Liu F.; Chen Y. Z.; Jiang Y. Predicting the Enzymatic Hydrolysis Half-lives of New Chemicals Using Support Vector Regression Models Based on Stepwise Feature Elimination. Molecular informatics 2017, 36, 1600153 10.1002/minf.201600153. 28627805

[ref29] Cerruela García G.; Pérez-Parras Toledano J.; de Haro García A.; García-Pedrajas N. Filter feature selectors in the development of binary QSAR models. SAR and QSAR in Environmental Research 2019, 30, 313–345. 10.1080/1062936X.2019.1588160. 31112077

[ref30] Cerruela García G.; García-Pedrajas N. Boosted feature selectors: a case study on prediction P-gp inhibitors and substrates. Journal of computer-aided molecular design 2018, 32, 1273–1294. 10.1007/s10822-018-0171-5. 30367310

[ref31] Antelo-Collado A.; Carrasco-Velar R.; García-Pedrajas N.; Cerruela-García G. Effective Feature Selection Method for Class-Imbalance Datasets Applied to Chemical Toxicity Prediction. Journal of Chemical Information and Modeling 2021, 61, 76–94. 10.1021/acs.jcim.0c00908. 33350301

[ref32] Bolón-Canedo V.; Alonso-Betanzos A. Ensembles for feature selection: A review and future trends. Information Fusion 2019, 52, 1–12. 10.1016/j.inffus.2018.11.008.

[ref33] Seijo-Pardo B.; Bolón-Canedo V.; Alonso-Betanzos A. On developing an automatic threshold applied to feature selection ensembles. Information Fusion 2019, 45, 227–245. 10.1016/j.inffus.2018.02.007.

[ref34] de Haro-García A.; Toledano J. P.-P.; Cerruela-García G.; García-Pedrajas N. Grab’Em: A Novel Graph-Based Method for Combining Feature Subset Selectors. IEEE Trans. Cybern. 2020,1 10.1109/TCYB.2020.3018815 33027013

[ref35] Skvortsova M.; Baskin I.; Skvortsov L.; Palyulin V.; Zefirov N.; Stankevich I. Chemical graphs and their basis invariants. J. Mol. Struct. 1999, 466, 211–217. 10.1016/S0166-1280(98)00467-9.

[ref36] Skvortsova M.; Fedyaev K.; Baskin I.; Palyulin V.; Zefirov N. A new technique for coding chemical structures based on basis fragments. Doklady Chemistry 2002, 382, 33–36. 10.1023/A:1014425222548.

[ref37] Li X.; Zhang Y.; Chen H.; Li H.; Zhao Y. In silico prediction of chronic toxicity with chemical category approaches. RSC Advances 2017, 7, 41330–41338. 10.1039/C7RA08415C.

[ref38] Schattel V.; Hinselmann G.; Jahn A.; Zell A.; Laufer S. Modeling and benchmark data set for the inhibition of c-Jun N-terminal kinase-3. Journal of chemical information and modeling 2011, 51, 670–679. 10.1021/ci100410h. 21280627

[ref39] Gramatica P. Computational Toxicology; Springer, 2013; pp 499–526.

[ref40] González-Medina M.; Prieto-Martínez F. D.; Owen J. R.; Medina-Franco J. L. Consensus diversity plots: a global diversity analysis of chemical libraries. J. Cheminf. 2016, 8, 63 10.1186/s13321-016-0176-9. PMC5105260 27895718

[ref41] Rogers D.; Hahn M. Extended-connectivity fingerprints. Journal of chemical information and modeling 2010, 50, 742–754. 10.1021/ci100050t. 20426451

[ref42] Landrum G. RDKit: Open-Source Cheminformatics Software. https://www.rdkit.org/docs/index.html (accessed Jun 16, 2021).

[ref43] Fontaine F.; Pastor M.; Zamora I.; Sanz F. Anchor- grind: Filling the gap between standard 3d qsar and the grid-independent descriptors. Journal of medicinal chemistry 2005, 48, 2687–2694. 10.1021/jm049113+. 15801859

[ref44] Hammann F.; Suenderhauf C.; Huwyler J. A binary ant colony optimization classifier for molecular activities. Journal of chemical information and modeling 2011, 51, 2690–2696. 10.1021/ci200186m. 21854036

[ref45] Ekins S.; Pottorf R.; Reynolds R. C.; Williams A. J.; Clark A. M.; Freundlich J. S. Looking back to the future: predicting in vivo efficacy of small molecules versus Mycobacterium tuberculosis. Journal of chemical information and modeling 2014, 54, 1070–1082. 10.1021/ci500077v. 24665947 PMC4004261

[ref46] Wu Z.; Ramsundar B.; Feinberg E. N.; Gomes J.; Geniesse C.; Pappu A. S.; Leswing K.; Pande V. MoleculeNet: a benchmark for molecular machine learning. Chemical science 2018, 9, 513–530. 10.1039/C7SC02664A. 29629118 PMC5868307

[ref47] Subramanian G.; Ramsundar B.; Pande V.; Denny R. A. Computational modeling of *β*-secretase 1 (BACE-1) inhibitors using ligand based approaches. Journal of chemical information and modeling 2016, 56, 1936–1949. 10.1021/acs.jcim.6b00290. 27689393

[ref48] Poongavanam V.; Haider N.; Ecker G. F. Fingerprint-based in silico models for the prediction of P-glycoprotein substrates and inhibitors. Bioorganic & medicinal chemistry 2012, 20, 5388–5395. 10.1016/j.bmc.2012.03.045. 22595422 PMC3445814

[ref49] Walker T.; Grulke C. M.; Pozefsky D.; Tropsha A. Chembench: a cheminformatics workbench. Bioinformatics 2010, 26, 3000–3001. 10.1093/bioinformatics/btq556. 20889496 PMC2982152

[ref50] Huuskonen J. Estimation of aqueous solubility for a diverse set of organic compounds based on molecular topology. J. Chem. Inf. Comput. Sci. 2000, 40, 773–777. 10.1021/ci9901338. 10850781

[ref51] Clark A. M.; Dole K.; Coulon-Spektor A.; McNutt A.; Grass G.; Freundlich J. S.; Reynolds R. C.; Ekins S. Open Source Bayesian Models. 1. Application to ADME/Tox and Drug Discovery Datasets. Journal of Chemical Information and Modeling 2015, 55, 1231–1245. 10.1021/acs.jcim.5b00143. 25994950 PMC4478615

[ref52] Perez-Rodriguez J.; de Haro-Garcia A.; Romero del Castillo J. A.; Garcia-Pedrajas N. A general framework for boosting feature subset selection algorithms. Information Fusion 2018, 44, 147–175. 10.1016/j.inffus.2018.03.003.

[ref53] Filzmoser P.; Liebmann B.; Varmuza K. Repeated double cross validation. Journal of Chemometrics: A Journal of the Chemometrics Society 2009, 23, 160–171. 10.1002/cem.1225.

[ref54] Ishibuchi H.; Nojima Y. Repeated double cross-validation for choosing a single solution in evolutionary multi-objective fuzzy classifier design. Knowledge-Based Systems 2013, 54, 22–31. 10.1016/j.knosys.2013.09.023.

[ref55] Tharwat A. Classification assessment methods. Appl. Comp. Inform. 2020, 17, 168 10.1016/j.aci.2018.08.003.

[ref56] Jo I.; Lee S.; Oh S. Improved measures of redundancy and relevance for mRMR feature selection. Computers 2019, 8, 42 10.3390/computers8020042.

[ref57] Demšar J. Statistical comparisons of classifiers over multiple data sets. J. Mach. Learn. Res. 2006, 7, 1–30.

[ref58] Nemenyi P. B. Distribution-Free Multiple Comparisons; Princeton University, 1963.

[ref59] Maudes J.; Rodríguez J. J.; García-Osorio C. Disturbing Neighbors Diversity for Decision Forests. In Applications of Supervised and Unsupervised Ensemble Methods; Springer, 2009; pp 113–133.

[ref60] Andersen M. E.; Krewski D. Toxicity Testing in the 21st Century: Bringing the Vision to Life. Toxicol. Sci. 2009, 107, 324–330. 10.1093/toxsci/kfn255. 19074763

[ref61] Krewski D.; Acosta D.; Andersen M.; Anderson H.; Bailar J. C.; Boekelheide K.; Brent R.; Charnley G.; Cheung V. G.; Green S.; Kelsey K. T.; Kerkvliet N. I.; Li A. A.; McCray L.; Meyer O.; Patterson R. D.; Pennie W.; Scala R. A.; Solomon G. M.; Stephens M.; Yager J.; Zeise L.; Toxicity Testing in the 21st Century: A Vision and a Strategy. Journal of Toxicology and Environmental Health, Part B 2010, 13, 51–138. 10.1080/10937404.2010.483176. PMC4410863 20574894

[ref62] Jiang J.; Wang R.; Wei G.-W. GGL-Tox: Geometric Graph Learning for Toxicity Prediction. Journal of chemical information and modeling 2021, 61, 1691–1700. 10.1021/acs.jcim.0c01294. 33719422 PMC8155789

