# Supplementary material for: Graph-Based Feature Selection Approach for Molecular Activity Prediction
Source: J Chem Inf Model. 2022 Mar 22;62(7):1618–32. doi: 10.1021/acs.jcim.1c01578 (PMC9006223; doi:10.1021/acs.jcim.1c01578)
Supplement: Supplementary file 1 — ci1c01578_si_001.pdf [file ci1c01578_si_001.pdf]

Supporting Information for Graph-based Feature Selection Approach for Molecular Activity Prediction

Gonzalo Cerruela-García,* José Manuel Cuevas-Muñoz, and Nicolás García-Pedrajas

University of Córdoba. Department of Computing and Numerical Analysis. Campus de Rabanales. Albert Einstein Building. E-14071 Córdoba, Spain.

E-mail: gcerruela@uco.es

- Tables S1 - S3: Experimental results in terms of *R*, *MI*, *AcRed*, *G-Mean* for each dataset.
- Table S4: TOX-21 benchmark description.
- Tables S5 - S7: Experimental results in terms of *R*, *MI*, *AcRed*, *G-Mean* for TOX-21 benchmark.
- Tables S8 - S9: Performance, reduction and redundancy statistical test results for TOX-21 benchmark.
- Figure S1: Distribution of physicochemical properties *ALogPS* and *MW* in each dataset, Figure S1 (a,b). The cumulative distribution function using the pairwise similarity values, Figure S1 (c).
- Figure S2: Experimental setup.

Table S 1: Performance results for DT classifier

Dataset	T				mTC				mT				MmTC				MmT			
	R	MI	AcRed	G-Mean	R	MI	AcRed	G-Mean	R	MI	AcRed	G-Mean	R	MI	AcRed	G-Mean	R	MI	AcRed	G-Mean
DS1	0.9279	0.4174	0.7546	0.7135	0.8554	0.2716	0.5487	0.8309	0.8522	0.2649	0.5348	0.8394	0.8552	0.2659	0.5404	0.8363	0.8561	0.2697	0.5451	0.8347
DS2	0.9146	0.1689	0.5118	0.0952	0.9014	0.152	0.4408	0.2835	0.9023	0.1379	0.4381	0.2608	0.8942	0.1336	0.4247	0.159	0.9007	0.1519	0.4464	0.2835
DS3	0.8691	0.4862	0.8233	0.7976	0.9803	0.4466	0.7911	0.8577	0.9666	0.4897	0.8003	0.8577	0.9717	0.4993	0.8069	0.8577	0.9629	0.4963	0.8294	0.851
DS4	0.8853	0.1905	0.4402	0.5354	0.9893	0.2614	0.5766	0.4214	0.9854	0.2705	0.5451	0.5598	0.9902	0.1809	0.421	0.4755	0.988	0.2852	0.5827	0.4443
DS5	0.8442	0.2155	0.56	0.6491	0.9641	0.3315	0.7043	0.6708	0.9772	0.3816	0.7648	0.6642	0.9668	0.2292	0.5937	0.6876	0.9761	0.3212	0.5758	0.5328
DS6	0.9137	0.4273	0.7269	0.6601	0.9888	0.3341	0.6272	0.6752	0.9882	0.3245	0.6175	0.7014	0.9861	0.347	0.6261	0.7025	0.9798	0.4015	0.6893	0.702
DS7	0.8838	0.4843	0.8007	0.6205	0.98	0.5541	0.8527	0.6616	0.9743	0.5498	0.8657	0.6552	0.9666	0.4013	0.6976	0.6243	0.9735	0.5324	0.8593	0.6525
DS8	0.8624	0.4141	0.7195	0.5936	0.9856	0.469	0.7033	0.6152	0.9784	0.6006	0.8927	0.6235	0.9856	0.5676	0.8631	0.616	0.9898	0.4004	0.6123	0.5907
DS9	0.9248	0.3757	0.569	0.7084	0.9283	0.2926	0.4971	0.7682	0.9343	0.3028	0.475	0.7633	0.9436	0.3467	0.5484	0.7575	0.9329	0.3186	0.5034	0.7543
DS10	0.9028	0.2135	0.5481	0.6137	0.9295	0.1728	0.4944	0.6518	0.9286	0.1757	0.4982	0.6768	0.9269	0.1816	0.4945	0.6409	0.9257	0.1758	0.4839	0.5985
DS11	0.8429	0.218	0.4505	0.5709	0.8893	0.2106	0.4319	0.6721	0.8859	0.2228	0.4363	0.6261	0.8889	0.2097	0.4271	0.6916	0.8868	0.2222	0.4435	0.7094
DS12	0.8814	0.5697	0.8203	0.706	0.8766	0.4684	0.7017	0.8132	0.8745	0.4547	0.6894	0.8023	0.8759	0.4921	0.7417	0.8025	0.8764	0.4931	0.7332	0.8253
DS13	0.9109	0.4638	0.8569	0.6669	0.942	0.3475	0.6953	0.7448	0.9392	0.3575	0.7317	0.7328	0.9394	0.3569	0.7205	0.7149	0.9378	0.3565	0.7058	0.7259
DS14	0.8539	0.3981	0.7291	0.5636	0.9921	0.5831	0.9108	0.6183	0.9682	0.5534	0.884	0.6205	0.9854	0.5443	0.8756	0.62	0.9782	0.5351	0.8397	0.6194
DS15	0.8987	0.2692	0.4656	0.8438	0.8856	0.2268	0.4641	0.8977	0.8808	0.2239	0.4517	0.8977	0.878	0.2148	0.4384	0.8977	0.8759	0.2222	0.4546	0.8977
DS16	0.9294	0.1912	0.4077	0.5273	0.994	0.1292	0.2627	0.3834	0.9946	0.2234	0.3893	0.4646	0.9946	0.2702	0.4616	0.4922	0.9931	0.1988	0.3614	0.4151
DS17	0.9004	0.1069	0.3369	0.5624	0.9794	0.1047	0.3241	0.5909	0.9947	0.1136	0.2956	0.5136	0.9926	0.1613	0.4244	0.5484	0.9893	0.1369	0.3605	0.561
DS18	0.8772	0.2243	0.5342	0.6514	0.9884	0.3587	0.6407	0.6609	0.9888	0.2842	0.5994	0.4302	0.99	0.2553	0.5821	0.5924	0.9914	0.1931	0.4307	0.6447
DS19	0.8468	0.1014	0.4325	0.5673	0.9868	0.1289	0.567	0.3462	0.9861	0.1561	0.5184	0.4365	0.9842	0.106	0.5811	0.4551	0.9801	0.1311	0.6374	0.452
DS20	0.9063	0.3296	0.7039	0.6471	0.9647	0.3032	0.645	0.6877	0.9573	0.2919	0.6274	0.689	0.9598	0.3174	0.6562	0.6884	0.9562	0.2901	0.6247	0.6886
DS21	0.8787	0.446	0.8396	0.5878	0.9065	0.3452	0.6886	0.6501	0.9074	0.3455	0.6865	0.6325	0.9065	0.3452	0.6874	0.6325	0.9125	0.3609	0.702	0.6498
DS22	0.9033	0.6441	0.9261	0.6383	0.9656	0.6383	0.8774	0.6763	0.9508	0.5859	0.8405	0.6761	0.9692	0.5809	0.8553	0.6786	0.9691	0.5735	0.8261	0.6791
DS23	0.8984	0.1878	0.5572	0.4802	0.9685	0.1852	0.5227	0.5053	0.9675	0.1934	0.5509	0.502	0.9661	0.191	0.5498	0.5049	0.9684	0.1567	0.4496	0.4717
DS24	0.9013	0.0732	0.2908	0.082	0.9069	0.0691	0.2694	0.0338	0.9023	0.0571	0.249	0.0338	0.9054	0.067	0.2625	0.0338	0.897	0.0673	0.2642	0.0338

Table S 2: Performance results for RF classifier

Dataset	T				mTC				mT				MmTC				MmT			
	R	MI	AcRed	G-Mean	R	MI	AcRed	G-Mean	R	MI	AcRed	G-Mean	R	MI	AcRed	G-Mean	R	MI	AcRed	G-Mean
DS1	0.9279	0.4174	0.7546	0.8086	0.8554	0.2716	0.5487	0.8918	0.8522	0.2649	0.5348	0.8969	0.8552	0.2659	0.5404	0.8972	0.8561	0.2697	0.5451	0.8985
DS2	0.9146	0.1689	0.5118	0.6143	0.9014	0.152	0.4408	0.6505	0.9023	0.1379	0.4381	0.6346	0.8942	0.1336	0.4247	0.6351	0.9007	0.1519	0.4464	0.6321
DS3	0.8691	0.4862	0.8233	0.8017	0.9803	0.4466	0.7911	0.8502	0.9666	0.4897	0.8003	0.8421	0.9717	0.4993	0.8069	0.8468	0.9629	0.4963	0.8294	0.8571
DS4	0.8853	0.1905	0.4402	0.6304	0.9893	0.2614	0.5766	0.4564	0.9854	0.2705	0.5451	0.6506	0.9902	0.1809	0.421	0.5073	0.988	0.2852	0.5827	0.4984
DS5	0.8442	0.2155	0.56	0.7353	0.9641	0.3315	0.7043	0.7608	0.9772	0.3816	0.7648	0.7344	0.9668	0.2292	0.5937	0.7632	0.9761	0.3212	0.5758	0.6103
DS6	0.9137	0.4273	0.7269	0.7373	0.9888	0.3341	0.6272	0.794	0.9882	0.3245	0.6175	0.7717	0.9861	0.347	0.6261	0.7817	0.9798	0.4015	0.6893	0.7917
DS7	0.8838	0.4843	0.8007	0.6382	0.98	0.5541	0.8527	0.6745	0.9743	0.5498	0.8657	0.6628	0.9666	0.4013	0.6976	0.6319	0.9735	0.5324	0.8593	0.6814
DS8	0.8624	0.4141	0.7195	0.6377	0.9856	0.469	0.7033	0.6368	0.9784	0.6006	0.8927	0.6449	0.9856	0.5676	0.8631	0.6568	0.9898	0.4004	0.6123	0.6211
DS9	0.9248	0.3757	0.569	0.7439	0.9283	0.2926	0.4971	0.8098	0.9343	0.3028	0.475	0.8072	0.9436	0.3467	0.5484	0.8004	0.9329	0.3186	0.5034	0.8042
DS10	0.9028	0.2135	0.5481	0.6131	0.9295	0.1728	0.4944	0.648	0.9286	0.1757	0.4982	0.663	0.9269	0.1816	0.4945	0.6435	0.9257	0.1758	0.4839	0.6545
DS11	0.8429	0.218	0.4505	0.6687	0.8893	0.2106	0.4319	0.7387	0.8859	0.2228	0.4363	0.7221	0.8889	0.2097	0.4271	0.695	0.8868	0.2222	0.4435	0.7319
DS12	0.8814	0.5697	0.8203	0.763	0.8766	0.4684	0.7017	0.7735	0.8745	0.4547	0.6894	0.7735	0.8759	0.4921	0.7417	0.7892	0.8764	0.4931	0.7332	0.7735
DS13	0.9109	0.4638	0.8569	0.7128	0.942	0.3475	0.6953	0.7823	0.9392	0.3575	0.7317	0.7756	0.9394	0.3569	0.7205	0.7665	0.9378	0.3565	0.7058	0.7744
DS14	0.8539	0.3981	0.7291	0.6621	0.9921	0.5831	0.9108	0.6506	0.9682	0.5534	0.884	0.6682	0.9854	0.5443	0.8756	0.6671	0.9782	0.5351	0.8397	0.6621
DS15	0.8987	0.2692	0.4656	0.8144	0.8856	0.2268	0.4641	0.8904	0.8808	0.2239	0.4517	0.9203	0.878	0.2148	0.4384	0.8946	0.8759	0.2222	0.4546	0.8992
DS16	0.9294	0.1912	0.4077	0.596	0.994	0.1292	0.2627	0.3832	0.9946	0.2234	0.3893	0.4955	0.9946	0.2702	0.4616	0.4632	0.9931	0.1988	0.3614	0.4412
DS17	0.9004	0.1069	0.3369	0.7093	0.9794	0.1047	0.3241	0.7441	0.9947	0.1136	0.2956	0.653	0.9926	0.1613	0.4244	0.7046	0.9893	0.1369	0.3605	0.7236
DS18	0.8772	0.2243	0.5342	0.7216	0.9884	0.3587	0.6407	0.7375	0.9888	0.2842	0.5994	0.7062	0.99	0.2553	0.5821	0.7656	0.9914	0.1931	0.4307	0.7368
DS19	0.8468	0.1014	0.4325	0.6775	0.9868	0.1289	0.567	0.4861	0.9861	0.1561	0.5184	0.5089	0.9842	0.106	0.5811	0.479	0.9801	0.1311	0.6374	0.5155
DS20	0.9063	0.3296	0.7039	0.7255	0.9647	0.3032	0.645	0.7756	0.9573	0.2919	0.6274	0.7764	0.9598	0.3174	0.6562	0.77	0.9562	0.2901	0.6247	0.7804
DS21	0.8787	0.446	0.8396	0.6879	0.9065	0.3452	0.6886	0.7692	0.9074	0.3455	0.6865	0.7743	0.9065	0.3452	0.6874	0.7697	0.9125	0.3609	0.702	0.7654
DS22	0.9033	0.6441	0.9261	0.7171	0.9656	0.6383	0.8774	0.7749	0.9508	0.5859	0.8405	0.7667	0.9692	0.5809	0.8553	0.7643	0.9691	0.5735	0.8261	0.7721
DS23	0.8984	0.1878	0.5572	0.6281	0.9685	0.1852	0.5227	0.6396	0.9675	0.1934	0.5509	0.6432	0.9661	0.191	0.5498	0.6551	0.9684	0.1567	0.4496	0.6161
DS24	0.9013	0.0732	0.2908	0.7038	0.9069	0.0691	0.2694	0.741	0.9023	0.0571	0.249	0.7518	0.9054	0.067	0.2625	0.7418	0.897	0.0673	0.2642	0.749

Table S 3: Performance results for SVM classifier

Dataset	T				mTC				mT				MmTC				MmT			
	R	MI	AcRed	G-Mean	R	MI	AcRed	G-Mean	R	MI	AcRed	G-Mean	R	MI	AcRed	G-Mean	R	MI	AcRed	G-Mean
DS1	0.9279	0.4174	0.7546	0.4687	0.8554	0.2716	0.5487	0.4921	0.8522	0.2649	0.5348	0.5045	0.8552	0.2659	0.5404	0.4917	0.8561	0.2697	0.5451	0.5045
DS2	0.9146	0.1689	0.5118	0.5639	0.9014	0.152	0.4408	0.4683	0.9023	0.1379	0.4381	0.5	0.8942	0.1336	0.4247	0.5221	0.9007	0.1519	0.4464	0.4797
DS3	0.8691	0.4862	0.8233	0.7827	0.9803	0.4466	0.7911	0.8407	0.9666	0.4897	0.8003	0.8498	0.9717	0.4993	0.8069	0.8479	0.9629	0.4963	0.8294	0.834
DS4	0.8853	0.1905	0.4402	0.6123	0.9893	0.2614	0.5766	0.4411	0.9854	0.2705	0.5451	0.5718	0.9902	0.1809	0.421	0.504	0.988	0.2852	0.5827	0.4672
DS5	0.8442	0.2155	0.56	0.6524	0.9641	0.3315	0.7043	0.7669	0.9772	0.3816	0.7648	0.738	0.9668	0.2292	0.5937	0.7653	0.9761	0.3212	0.5758	0.5753
DS6	0.9137	0.4273	0.7269	0.7128	0.9888	0.3341	0.6272	0.7422	0.9882	0.3245	0.6175	0.7396	0.9861	0.347	0.6261	0.7461	0.9798	0.4015	0.6893	0.7494
DS7	0.8838	0.4843	0.8007	0.6088	0.98	0.5541	0.8527	0.6391	0.9743	0.5498	0.8657	0.6775	0.9666	0.4013	0.6976	0.6197	0.9735	0.5324	0.8593	0.67
DS8	0.8624	0.4141	0.7195	0.5572	0.9856	0.469	0.7033	0.6723	0.9784	0.6006	0.8927	0.6599	0.9856	0.5676	0.8631	0.6849	0.9898	0.4004	0.6123	0.537
DS9	0.9248	0.3757	0.569	0.7115	0.9283	0.2926	0.4971	0.7446	0.9343	0.3028	0.475	0.738	0.9436	0.3467	0.5484	0.7493	0.9329	0.3186	0.5034	0.7482
DS10	0.9028	0.2135	0.5481	0.6213	0.9295	0.1728	0.4944	0.6238	0.9286	0.1757	0.4982	0.6533	0.9269	0.1816	0.4945	0.6507	0.9257	0.1758	0.4839	0.6546
DS11	0.8429	0.218	0.4505	0.342	0.8893	0.2106	0.4319	0.5689	0.8859	0.2228	0.4363	0.5136	0.8889	0.2097	0.4271	0.5358	0.8868	0.2222	0.4435	0.5136
DS12	0.8814	0.5697	0.8203	0.7418	0.8766	0.4684	0.7017	0.7357	0.8745	0.4547	0.6894	0.7357	0.8759	0.4921	0.7417	0.7061	0.8764	0.4931	0.7332	0.7357
DS13	0.9109	0.4638	0.8569	0.7363	0.942	0.3475	0.6953	0.8078	0.9392	0.3575	0.7317	0.7859	0.9394	0.3569	0.7205	0.8006	0.9378	0.3565	0.7058	0.7988
DS14	0.8539	0.3981	0.7291	0.6277	0.9921	0.5831	0.9108	0.6363	0.9682	0.5534	0.884	0.6524	0.9854	0.5443	0.8756	0.6696	0.9782	0.5351	0.8397	0.657
DS15	0.8987	0.2692	0.4656	0.3278	0.8856	0.2268	0.4641	0.1894	0.8808	0.2239	0.4517	0.2701	0.878	0.2148	0.4384	0.2471	0.8759	0.2222	0.4546	0.1745
DS16	0.9294	0.1912	0.4077	0.5843	0.994	0.1292	0.2627	0.3821	0.9946	0.2234	0.3893	0.4662	0.9946	0.2702	0.4616	0.4794	0.9931	0.1988	0.3614	0.4337
DS17	0.9004	0.1069	0.3369	0.6748	0.9794	0.1047	0.3241	0.7103	0.9947	0.1136	0.2956	0.6159	0.9926	0.1613	0.4244	0.6517	0.9893	0.1369	0.3605	0.6871
DS18	0.8772	0.2243	0.5342	0.5971	0.9884	0.3587	0.6407	0.6849	0.9888	0.2842	0.5994	0.6946	0.99	0.2553	0.5821	0.7135	0.9914	0.1931	0.4307	0.6908
DS19	0.8468	0.1014	0.4325	0.4002	0.9868	0.1289	0.567	0.4479	0.9861	0.1561	0.5184	0.3592	0.9842	0.106	0.5811	0.4549	0.9801	0.1311	0.6374	0.4671
DS20	0.9063	0.3296	0.7039	0.6745	0.9647	0.3032	0.645	0.75	0.9573	0.2919	0.6274	0.7434	0.9598	0.3174	0.6562	0.7492	0.9562	0.2901	0.6247	0.7355
DS21	0.8787	0.446	0.8396	0.6002	0.9065	0.3452	0.6886	0.5711	0.9074	0.3455	0.6865	0.5668	0.9065	0.3452	0.6874	0.5668	0.9125	0.3609	0.702	0.5789
DS22	0.9033	0.6441	0.9261	0.6881	0.9656	0.6383	0.8774	0.7501	0.9508	0.5859	0.8405	0.7347	0.9692	0.5809	0.8553	0.7239	0.9691	0.5735	0.8261	0.7299
DS23	0.8984	0.1878	0.5572	0.5501	0.9685	0.1852	0.5227	0.5784	0.9675	0.1934	0.5509	0.5836	0.9661	0.191	0.5498	0.5875	0.9684	0.1567	0.4496	0.5371
DS24	0.9013	0.0732	0.2908	0.607	0.9069	0.0691	0.2694	0.6082	0.9023	0.0571	0.249	0.6223	0.9054	0.067	0.2625	0.6121	0.897	0.0673	0.2642	0.6259

Table S 4: Toxicity Datasets (TOX-21)

Dataset	Molecular pathway endpoint
TOX1	Androgen receptor MDA-kb2 AR-luc cell line (NR-AR)
TOX2	Androgen receptor GeneBLAzer AR-UAS-bla-GripTite cell line (NR-AR-LBD)
TOX3	Aryl hydrocarbon receptor (NR-AhR)
TOX4	Aromatase enzyme (NR-Aromatase)
TOX5	Estrogen receptor alpha BG1-Luc-4E2 cell line (NR-ER)
TOX6	Estrogen receptor alpha ER-alpha-UAS-bla GripTiteTM cell line (NR-ER-LBD)
TOX7	Peroxisome proliferator-activated receptor gamma (NR-PPAR-gamma)
TOX8	Nuclear factor (erythroid-derived 2)-like 2/antioxidant responsive element (NR-ARE) (SR-ARE)
TOX9	ATAD5 receptor (SR-ATAD5)
TOX10	Heat shock factor response element (SR-HSE)
TOX11	Mitochondrial membrane potential (SR-MMP)
TOX12	p53 signaling pathway (SR-p53)

Table S 5: TOX-21 Results for DT classifier

Dataset	T				mTC				mT				MmTC				MmT			
	R	MI	AcRed	G-Mean	R	MI	AcRed	G-Mean	R	MI	AcRed	G-Mean	R	MI	AcRed	G-Mean	R	MI	AcRed	G-Mean
TOX1	0.9483	0.3127	0.7163	0.2858	0.9921	0.2164	0.5607	0.3557	0.9917	0.2446	0.5638	0.3823	0.9794	0.2467	0.6265	0.5307	0.9875	0.2058	0.5478	0.3508
TOX2	0.9417	0.1684	0.378	0.2036	0.9861	0.2433	0.5882	0.1922	0.9875	0.2457	0.6024	0.2892	0.9919	0.2338	0.5823	0.2716	0.9877	0.1986	0.5353	0.2367
TOX3	0.9258	0.7421	0.9052	0.2028	0.9712	0.3282	0.6953	0.2375	0.968	0.2967	0.6461	0.2932	0.9803	0.3181	0.6923	0.4235	0.9793	0.3001	0.6534	0.3323
TOX4	0.925	0.699	0.958	0.1732	0.9499	0.5926	0.8517	0.1823	0.9735	0.5856	0.8283	0.2634	0.9455	0.5543	0.7976	0.369	0.9506	0.5026	0.754	0.029
TOX5	0.945	0.5171	0.833	0.1743	0.9891	0.4776	0.8023	0.2679	0.988	0.4891	0.7828	0.2735	0.9863	0.4127	0.706	0.3887	0.9851	0.4187	0.7319	0.3476
TOX6	0.9223	0.4064	0.8136	0.2268	0.9705	0.3872	0.7303	0.1823	0.9789	0.3078	0.6843	0.2437	0.9789	0.3458	0.7191	0.4327	0.9808	0.3046	0.6591	0.3632
TOX7	0.9255	0.1443	0.4786	0.1603	0.9661	0.2513	0.5477	0.1867	0.9798	0.1056	0.4887	0.2635	0.9782	0.1685	0.49	0.2854	0.9696	0.0357	0.3272	0.2158
TOX8	0.9316	0.4971	0.9041	0.1846	0.9858	0.4731	0.8082	0.2146	0.9888	0.4091	0.8075	0.2785	0.9868	0.4746	0.836	0.3988	0.9831	0.4338	0.8061	0.2965
TOX9	0.9238	0.1455	0.4784	0.1396	0.9548	0.1814	0.4929	0.1258	0.9735	0.039	0.4166	0.1716	0.9568	0.1497	0.5393	0.273	0.9654	0.112	0.4106	0.2161
TOX10	0.8722	0.035	0.3231	0.1515	0.8924	0.0368	0.2777	0.1654	0.9374	0.0356	0.3211	0.1921	0.916	0.0319	0.2986	0.1961	0.9083	0.0265	0.2462	0.1999
TOX11	0.881	0.4868	0.7802	0.2098	0.9603	0.5769	0.8406	0.1551	0.9793	0.5771	0.865	0.1257	0.9373	0.5305	0.7879	0.2193	0.9575	0.4815	0.7768	0.1768
TOX12	0.9199	0.5151	0.9542	0.1659	0.968	0.4395	0.8756	0.1595	0.981	0.447	0.8721	0.2477	0.9873	0.4612	0.9008	0.3446	0.9798	0.4595	0.8957	0.1595

Table S 6: TOX-21 Results for RF classifier

Dataset	T				mTC				mT				MmTC				MmT			
	R	MI	AcRed	G-Mean	R	MI	AcRed	G-Mean	R	MI	AcRed	G-Mean	R	MI	AcRed	G-Mean	R	MI	AcRed	G-Mean
TOX1	0.9483	0.3127	0.7163	0.6329	0.9921	0.2164	0.5607	0.6455	0.9917	0.2446	0.5638	0.6629	0.9794	0.2467	0.6265	0.6504	0.9875	0.2058	0.5478	0.6553
TOX2	0.9417	0.1684	0.378	0.6335	0.9861	0.2433	0.5882	0.6729	0.9875	0.2457	0.6024	0.6942	0.9919	0.2338	0.5823	0.6909	0.9877	0.1986	0.5353	0.6888
TOX3	0.9258	0.7421	0.9052	0.1965	0.9712	0.3282	0.6953	0.4828	0.968	0.2967	0.6461	0.4864	0.9803	0.3181	0.6923	0.4947	0.9793	0.3001	0.6534	0.4999
TOX4	0.925	0.699	0.958	0.5742	0.9499	0.5926	0.8517	0.361	0.9735	0.5856	0.8283	0.3678	0.9455	0.5543	0.7976	0.3801	0.9506	0.5026	0.754	0.3664
TOX5	0.945	0.5171	0.833	0.4196	0.9891	0.4776	0.8023	0.4396	0.988	0.4891	0.7828	0.4401	0.9863	0.4127	0.706	0.4463	0.9851	0.4187	0.7319	0.4506
TOX6	0.9223	0.4064	0.8136	0.4074	0.9705	0.3872	0.7303	0.4536	0.9789	0.3078	0.6843	0.4641	0.9789	0.3458	0.7191	0.4631	0.9808	0.3046	0.6591	0.486
TOX7	0.9255	0.1443	0.4786	0.2042	0.9661	0.2513	0.5477	0.2045	0.9798	0.1056	0.4887	0.094	0.9782	0.1685	0.49	0.2076	0.9696	0.0357	0.3272	0.1711
TOX8	0.9316	0.4971	0.9041	0.3546	0.9858	0.4731	0.8082	0.3655	0.9888	0.4091	0.8075	0.3735	0.9868	0.4746	0.836	0.3719	0.9831	0.4338	0.8061	0.3604
TOX9	0.9238	0.1455	0.4784	0.2751	0.9548	0.1814	0.4929	0.2626	0.9735	0.039	0.4166	0.3128	0.9568	0.1497	0.5393	0.277	0.9654	0.112	0.4106	0.2752
TOX10	0.8722	0.035	0.3231	0.2883	0.8924	0.0368	0.2777	0.2852	0.9374	0.0356	0.3211	0.2509	0.916	0.0319	0.2986	0.2722	0.9083	0.0265	0.2462	0.2767
TOX11	0.881	0.4868	0.7802	0.4907	0.9603	0.5769	0.8406	0.5142	0.9793	0.5771	0.865	0.5059	0.9373	0.5305	0.7879	0.5175	0.9575	0.4815	0.7768	0.532
TOX12	0.9199	0.5151	0.9542	0.2937	0.968	0.4395	0.8756	0.3027	0.981	0.447	0.8721	0.2951	0.9873	0.4612	0.9008	0.3077	0.9798	0.4595	0.8957	0.2967

Table S 7: TOX-21 Results for SVM classifier

Dataset	T				mTC				mT				MmTC				MmT			
	R	MI	AcRed	G-Mean	R	MI	AcRed	G-Mean	R	MI	AcRed	G-Mean	R	MI	AcRed	G-Mean	R	MI	AcRed	G-Mean
TOX1	0.9483	0.3127	0.7163	0.6442	0.9921	0.2164	0.5607	0.6534	0.9917	0.2446	0.5638	0.6376	0.9794	0.2467	0.6265	0.6042	0.9875	0.2058	0.5478	0.6242
TOX2	0.9417	0.1684	0.378	0.619	0.9861	0.2433	0.5882	0.6398	0.9875	0.2457	0.6024	0.6378	0.9919	0.2338	0.5823	0.6833	0.9877	0.1986	0.5353	0.6696
TOX3	0.9258	0.7421	0.9052	0.3903	0.9712	0.3282	0.6953	0.3764	0.968	0.2967	0.6461	0.4112	0.9803	0.3181	0.6923	0.3543	0.9793	0.3001	0.6534	0.3832
TOX4	0.925	0.699	0.958	0.0	0.9499	0.5926	0.8517	0.136	0.9735	0.5856	0.8283	0.0947	0.9455	0.5543	0.7976	0.1517	0.9506	0.5026	0.754	0.1698
TOX5	0.945	0.5171	0.833	0.3055	0.9891	0.4776	0.8023	0.3057	0.988	0.4891	0.7828	0.3209	0.9863	0.4127	0.706	0.3232	0.9851	0.4187	0.7319	0.3301
TOX6	0.9223	0.4064	0.8136	0.3925	0.9705	0.3872	0.7303	0.4086	0.9789	0.3078	0.6843	0.4416	0.9789	0.3458	0.7191	0.4269	0.9808	0.3046	0.6591	0.4363
TOX7	0.9255	0.1443	0.4786	0.0764	0.9661	0.2513	0.5477	0.0943	0.9798	0.1056	0.4887	0.0333	0.9782	0.1685	0.49	0.0943	0.9696	0.0357	0.3272	0.0471
TOX8	0.9316	0.4971	0.9041	0.1625	0.9858	0.4731	0.8082	0.2126	0.9888	0.4091	0.8075	0.1612	0.9868	0.4746	0.836	0.2172	0.9831	0.4338	0.8061	0.1867
TOX9	0.9238	0.1455	0.4784	0.0524	0.9548	0.1814	0.4929	0.0277	0.9735	0.039	0.4166	0.0277	0.9568	0.1497	0.5393	0.0829	0.9654	0.112	0.4106	0.0552
TOX10	0.8722	0.035	0.3231	0.0603	0.8924	0.0368	0.2777	0.0796	0.9374	0.0356	0.3211	0.0965	0.916	0.0319	0.2986	0.0731	0.9083	0.0265	0.2462	0.122
TOX11	0.881	0.4868	0.7802	0.36	0.9603	0.5769	0.8406	0.4033	0.9793	0.5771	0.865	0.3446	0.9373	0.5305	0.7879	0.412	0.9575	0.4815	0.7768	0.3622
TOX12	0.9199	0.5151	0.9542	0.1194	0.968	0.4395	0.8756	0.1128	0.981	0.447	0.8721	0.0839	0.9873	0.4612	0.9008	0.1279	0.9798	0.4595	0.8957	0.1279

Table S 8: Performance (*G-Mean*) statistical tests results for TOX-21 datasets †

	T	mT	MmT	mTC	MmTC
**DT** (Iman-Davenport=0.0000, Nemenyi CD= 1.7609)					
Mean	0.1899	0.2520	0.2437	0.2021	0.3444
Ranks	4.2500	2.6667	2.7917	4.1250	1.1667
Holm p-values	0.0000	0.0101	0.0059	0.0000	-
Holm thresholds	0.0125	0.0500	0.0250	0.0167	-
Holm test	+	+	+	+	👍
**RF** (Iman-Davenport=0.0138, Nemenyi CD= 1.7609)					
Mean	0.3976	0.4123	0.4216	0.4159	0.4233
Ranks	4.0833	2.7500	2.5000	3.5000	2.1667
Holm p-values	0.0015	0.1831	0.3028	0.0194	-
Holm thresholds	0.0125	0.0250	0.0500	0.0167	-
Holm test	+	=	=	=	👍
**SVM** (Iman-Davenport=0.046826, Nemenyi CD= 1.7609)					
Mean	0.2652	0.2743	0.2929	0.2875	0.2959
Ranks	3.8333	3.5833	2.3333	2.9167	2.3333
Holm p-values	0.0101	0.0264	-	0.1831	-
Holm thresholds	0.0167	0.0250	-	0.0500	-
Holm test	+	=	👍	=	👍

† The best method according to the Holm test is indicated by the symbol "👍"

Table S 9: Reduction and redundancy statistical tests results for TOX-21 datasets †

R
(Iman-Davenport=0.0000, Nemenyi CD= 1.7609)

	T	mT	MmT	mTC	MmTC
Mean	0.9218	0.9773	0.9696	0.9655	0.9687
Ranks	5.0000	1.8333	2.6667	3.0833	2.4167
Holm p-values	0.0000	-	0.0984	0.0264	0.1831
Holm thresholds	0.0125	-	0.0250	0.0167	0.0500
Holm test	+	👍	=	=	=

MI
(Iman-Davenport=0.0010, Nemenyi CD= 1.7609)

	T	mT	MmT	mTC	MmTC
Mean	0.3891	0.3153	0.2900	0.3504	0.3273
Ranks	3.9167	2.7500	1.5833	3.6667	3.0833
Holm p-values	0.0002	0.0354	-	0.0006	0.0101
Holm thresholds	0.0125	0.0500	-	0.0167	0.0250
Holm test	+	+	👍	+	+

AcRed
(Iman-Davenport=0.0002, Nemenyi CD= 1.7609)

	T	mT	MmT	mTC	MmTC
Mean	0.7102	0.6566	0.6120	0.6726	0.6647
Ranks	4.0000	2.8333	1.4167	3.5000	3.2500
Holm p-values	0.0000	0.0141	-	0.0006	0.0023
Holm thresholds	0.0125	0.0500	-	0.0167	0.0250
Holm test	+	+	👍	+	+

† The best method according to the Holm test is indicated by the symbol "👍"

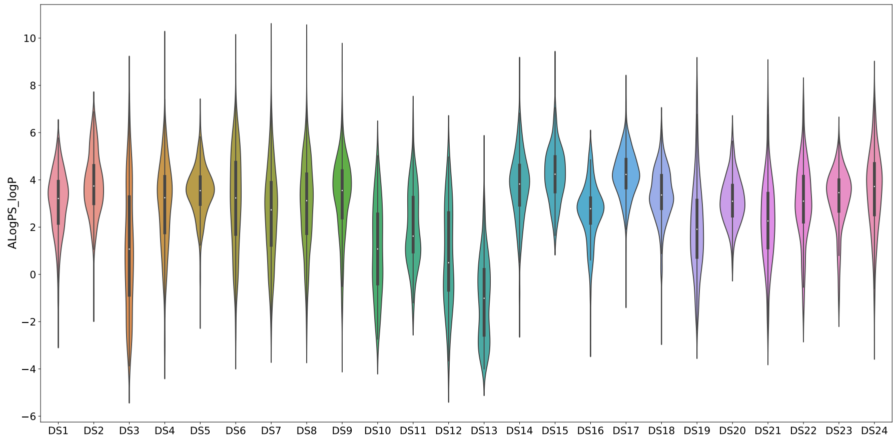

(a) Violin plot for octanol/water partition coefficient

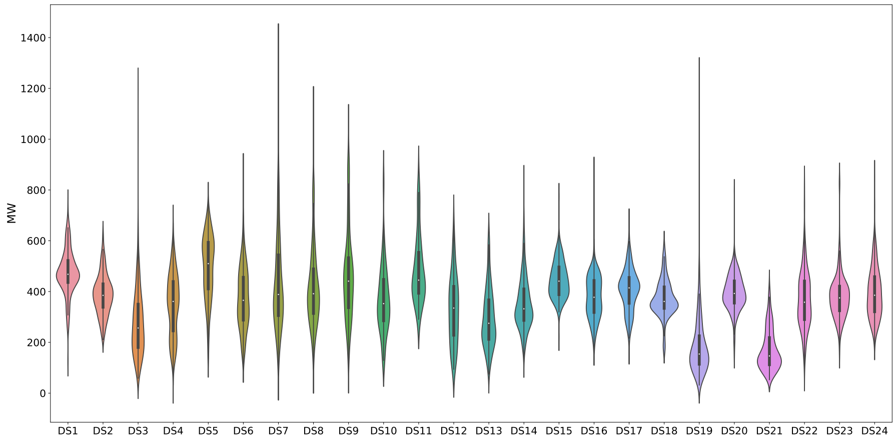

(b) Violin plot for Molecular Weight

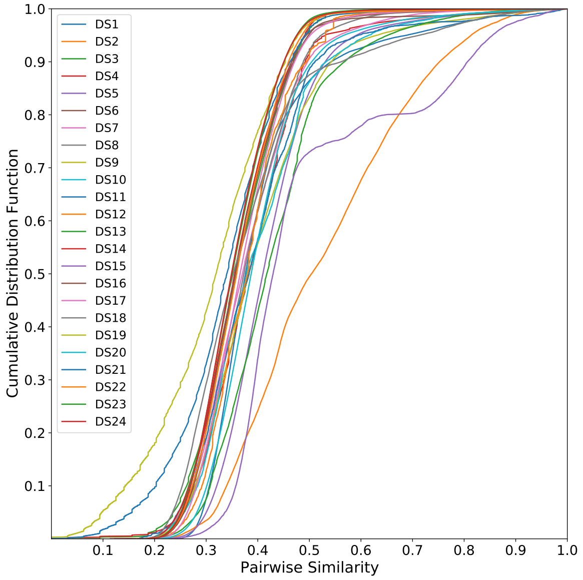

(c) Similarity cumulative distribution function

Figure S 1: Property and similarity diversity of the datasets

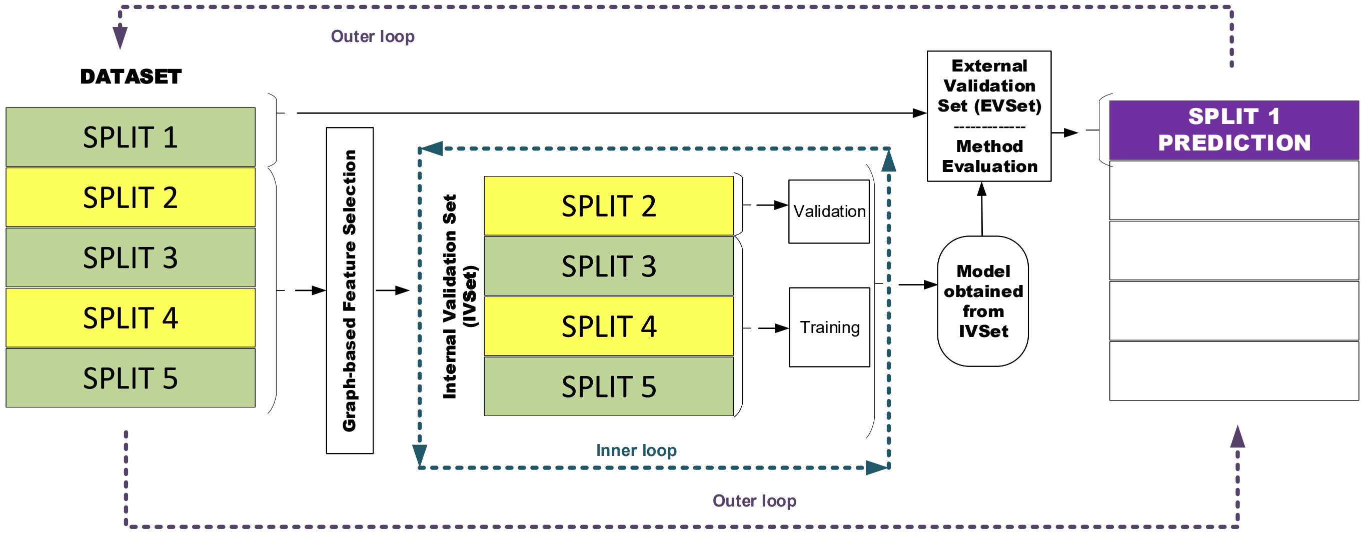

Figure S 2: Experimental setup
